# Triple RNA-Seq characterizes aphid gene expression in response to infection with unequally virulent strains of the endosymbiont *Hamiltonella defensa*

**DOI:** 10.1186/s12864-021-07742-8

**Published:** 2021-06-16

**Authors:** Heidi Kaech, Alice B. Dennis, Christoph Vorburger

**Affiliations:** 1 Aquatic Ecology, Eawag, Swiss Federal Institute of Aquatic Science and Technology, Dübendorf, Switzerland; 2 D-USYS, Department of Environmental Systems Science, ETH Zürich, Zürich, Switzerland; 3 Institute of Biochemistry and Biology, University Potsdam, Potsdam, Germany

**Keywords:** *Aphis fabae*, *Buchnera*, Cost of resistance, *Hamiltonella*, Host-symbiont interaction, RNA-Seq, Symbiosis

## Abstract

**Background:**

Secondary endosymbionts of aphids provide benefits to their hosts, but also impose costs such as reduced lifespan and reproductive output. The aphid *Aphis fabae* is host to different strains of the secondary endosymbiont *Hamiltonella defensa*, which encode different putative toxins. These strains have very different phenotypes: They reach different densities in the host, and the costs and benefits (protection against parasitoid wasps) they confer to the host vary strongly.

**Results:**

We used RNA-Seq to generate hypotheses on why four of these strains inflict such different costs to *A. fabae*. We found different *H. defensa* strains to cause strain-specific changes in aphid gene expression, but little effect of *H. defensa* on gene expression of the primary endosymbiont, *Buchnera aphidicola*. The highly costly and over-replicating *H. defensa* strain H85 was associated with strongly reduced aphid expression of hemocytin, a marker of hemocytes in *Drosophila*. The closely related strain H15 was associated with downregulation of ubiquitin-related modifier 1, which is related to nutrient-sensing and oxidative stress in other organisms. Strain H402 was associated with strong differential regulation of a set of hypothetical proteins, the majority of which were only differentially regulated in presence of H402.

**Conclusions:**

Overall, our results suggest that costs of different strains of *H. defensa* are likely caused by different mechanisms, and that these costs are imposed by interacting with the host rather than the host’s obligatory endosymbiont *B. aphidicola*.

**Supplementary Information:**

The online version contains supplementary material available at 10.1186/s12864-021-07742-8.

## Background

Insects have a complex evolutionary history with bacteria. On one hand, they are exposed to environmental bacterial pathogens, against which their immune system should defend them [1]. On the other hand, insects commonly harbour beneficial bacterial endosymbionts, which their immune system should tolerate [2]. In aphids, tolerance of the primary bacterial endosymbiont *Buchnera aphidicola* is necessary for survival, as *B. aphidicola* supplements the aphids’ protein-poor diet with essential amino acids [3–6]. This ancient symbiosis, which is at least 160 Ma old [4], may be facilitated by the seclusion of *B. aphidicola* to specialized bacteriocytes [2]. *Buchnera aphidicola* is vertically transmitted from mother to offspring [7].

Aphids also maintain a range of secondary bacterial endosymbionts. Like *B. aphidicola*, these secondary endosymbionts provide benefits, are vertically transmitted, and some of them can be found intracellularly [8, 9]. Unlike *B. aphidicola*, however, they are not strictly required for survival and also colonise the extracellular space [9]. In fact, their density in the hemolymph is sufficiently high to allow horizontal transmission to other aphids, both via artificial microinjection of hemolymph, naturally via vectors such as parasitoid wasps [10], or via host plants [11].

The continuous presence of secondary endosymbionts in the hemolymph suggests that the aphids’ immune system allows their presence. Maintenance of secondary endosymbionts might partially be attributable to peculiarities of the aphids’ immune system. Comparative genomics of *Drosophila melanogaster* and the pea aphid, *Acyrthosiphon pisum,* suggest a reduced immune system repertoire in the latter. In the pea aphid, one of the two humoral response pathways, the immune deficiency (IMD) pathway, which is preferentially activated by Gram-negative bacteria in *Drosophila* [12], lacks several key proteins and pattern recognition receptors [13]. It was proposed that this facilitated the association of aphids with their mostly Gram-negative endosymbionts [14, 15]. In support of this, pea aphids react strongly to heat-killed fungi, but only weakly to heat-killed Gram-negative pathogens [13, 16], and experimental infection with Gram-negative *Escherichia coli* is fatal to pea aphids [17]. Yet, the immune response to Gram-negative bacteria may be inefficient in aphids, but it is not non-existent; in response to infection with *Serratia marcescens,* pea aphids mount a seemingly IMD-independent activation of the c-Jun N-terminal kinase (JNK) pathway [18] and upon challenge with *E. coli,* hemocytes readily destroy *E. coli* through phagocytosis [14, 19]. Secondary symbionts might have to protect themselves from these immune responses to allow stable association with their host.

The amount of endosymbionts that a host possesses (measured as titre) may influence host fitness, as secondary endosymbionts provide benefits to their hosts, but could also be deleterious if they proliferated uncontrollably. Benefits of secondary symbionts include defence against pathogens [20], protection from parasitoids [21], adaptation to host plants [22], and heat shock tolerance [23]. Despite these benefits, secondary endosymbionts only occur at intermediate frequencies in aphid populations [24, 25]. Their spread through the host populations appears to be constrained by costs, which are apparent when populations of the same aphid genotype with and without secondary endosymbionts compete against each other in experimental populations [26–28]. If secondary endosymbionts are inherently costly, the host should profit from controlling their density so that the optimal balance between their costs and benefits is achieved. Whether such control exists in aphids and how it might be achieved – for example through special seclusion and metabolic control [29–31] – is yet unknown.

A frequent secondary endosymbiont of aphids is *Hamiltonella defensa*. It provides protection against aphid parasitoids such as *Aphidius ervi* [32] and *Lysiphlebus fabarum* [33, 34]. While *H. defensa* itself encodes putative toxins that could potentially hinder parasitoid development, the strongest link to its protective function is with the lysogenic bacteriophage APSE (*A. pisum* secondary endosymbiont) [35, 36]. This phage is integrated in the *H. defensa* genome and occurs in variants that encode different putative toxins [37, 38]. Spontaneous loss of APSE in strains hosted by pea aphids is associated with the loss of protection against parasitoids and over-replication of *H. defensa* [36, 39]. In the black bean aphid (*Aphis fabae*), *H. defensa* and its associated APSE lead to a reduced lifespan and lifetime reproduction in the absence of parasitoids [40]. Possible explanations include the resource consumption by the endosymbiont population, collateral damage to the host from the APSE’s toxins, or the energy requirements of immune activation if secondary endosymbionts have to be controlled by the aphid’s immune system [41].

**Fig. 1:**
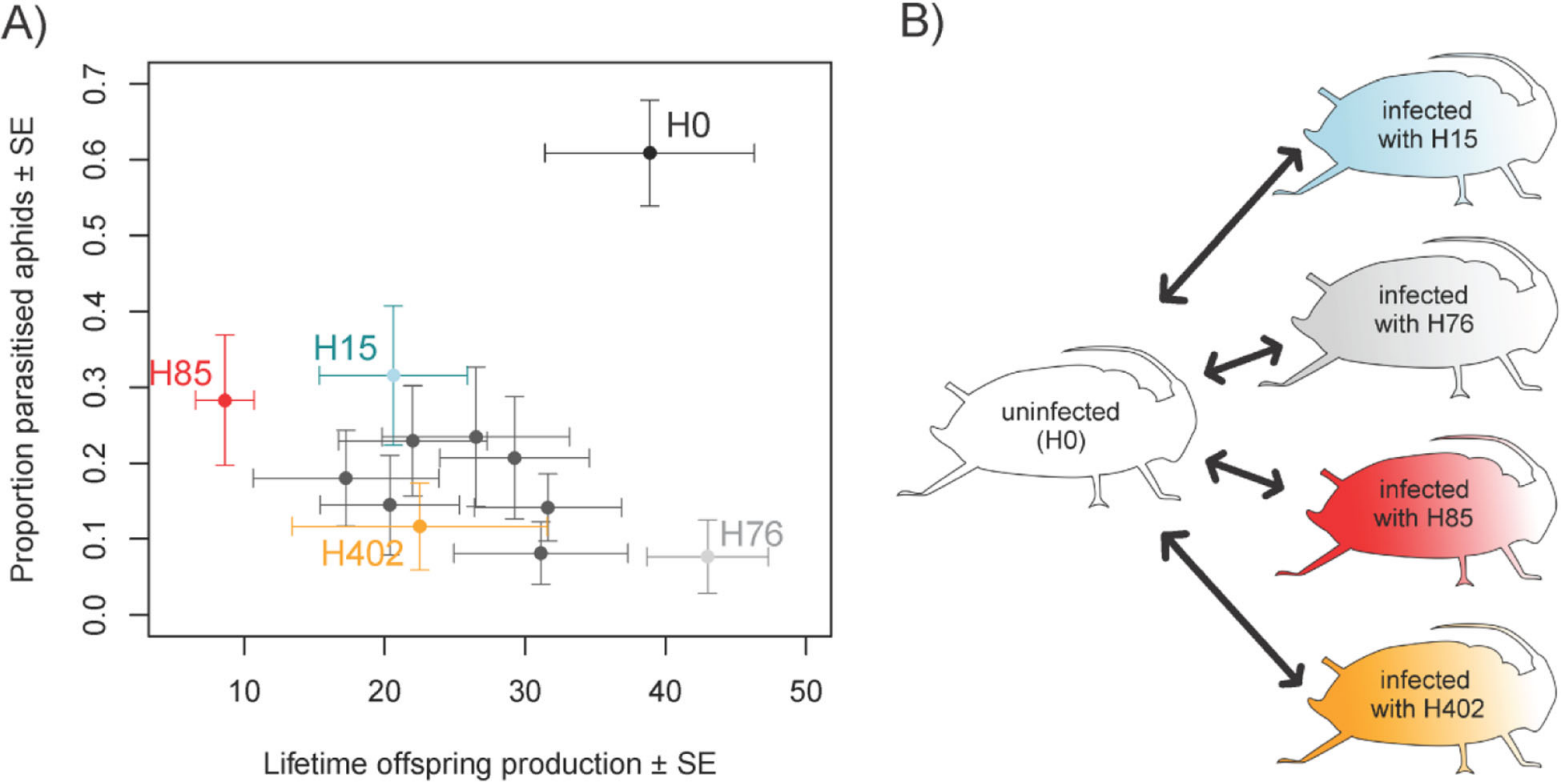
Properties of different *H. defensa* strains and experimental design. **A** Effect of different *H. defensa* strains on lifetime offspring production (cost) and susceptibility to parasitism (benefit) of black bean aphids, *Aphis fabae* (adapted from Cayetano et al. 2015). Aphids belonged to a single clone (A06–407) and were either uninfected (H0) or infected with different *H. defensa* strains. Strains that we used in this experiment are marked in colour: H15 (blue), H402 (orange), H76 (grey) and H85 (red). **B** Our experiment compares gene expression between sublines of the aphid clone A06–407 infected by *H. defensa* (infecting strains: H15, H402, H76 or H85) and the uninfected subline (H0)

For *H. defensa* in black bean aphids, Cayetano et al. [42] showed in a comparison of 11 strains, that some strains strongly protect hosts against parasitation by *L. fabarum* but have little impact on host longevity and offspring production, while others are more weakly protective but highly costly (Fig. 1 A). In this work, we investigate four *H. defensa* strains that were part of the experiment of Cayetano et al. [42]: H15, H76, H85 and H402. These were chosen to represent different APSE toxin cassettes [43] and to span the known haplotypes of *H. defensa* in *A. fabae*. Strain H76 belongs to the *H. defensa* haplotype 1. It carries an APSE that encodes a YD-repeat toxin gene with two open reading frames (NCBI GenBank: KU175898). Cayetano et al. [42] found that the protection against the parasitoid *Lysiphlebus fabarum* provided by H76 is very strong, while aphids infected by H76 were virtually as fecund as uninfected controls (Fig. 1 A). Strain H402 belongs to haplotype 2. It carries an APSE that encodes a CdtB-toxin (NCBI GenBank: KU175897). The protection provided by H402 is intermediate, and so are its costs [42] (Fig. 1 A). Strains H15 and H85 belong to haplotype 3, provide limited protection and entail high costs (Fig. 1 A) [42]. H85 carries an APSE encoding a YD-repeat toxin gene that is longer than the one of H76, while for H15 the APSE toxin was not sequenced prior to this experiment. Strain H85 is particularly costly: Aphids infected with H85 die shortly after reaching adulthood. In contrast to H15, H85 reaches very high density in the host [42, 44]. The four strains thus have very different phenotypes: from the mutualistic benefits conferred by H76 to the over-replicating and costly H85, which behaves more like a pathogen.

In this work, we have employed ‘triple’ RNA-Seq to measure gene expression of *A. fabae* and their obligate endosymbiont *B. aphidicola* in the presence or absence of different strains of the secondary endosymbiont *H. defensa* (Fig. 1 B)*.* We have used this to generate hypotheses about how different *H. defensa* strains inflict costs on the black bean aphid host and whether the host regulates the density of *H. defensa*.

## Results

### Sequencing output

We sequenced the transcriptome of aphids carrying only their obligatory endosymbiont *B. aphidicola* (H0) and identically reared aphids from the same genetic background infected by one out of four different *H. defensa* strains: H15, H76, H85 or H402. Each of the five treatment was replicated four times (R1-R4). One of the 20 libraries, library H15R1, was heavily contaminated with reads of human and human-associated bacterial origin (Supplementary Table 2). This library also took an outlier position in a PCA built from overall aphid gene expression patterns (Supplementary Fig. 1) and was therefore excluded from further analyses. Our approach could be called a ‘triple’ RNA-Seq because it contains transcripts from three organisms – aphid host, obligatory endosymbiont and secondary endosymbiont.

### Assembly

For aphids, the assembly generated 46′352 transcripts. Transcript length ranged from 297 to 27′541 nucleotides (mean length: 2′657.9 bp, N_50_: 3′542 bp, GC: 32.02%). Transcripts were assigned by blast to a total of 10′809 genes, of which 7′313 could be annotated with GO-terms. In comparison, the genome of *Aphis glycines* contains 17′558 genes [45]. In our assembly, 93.1% of the *Insecta* BUSCO genes were complete, while 2.6% were fragmented (Supplementary Table 3).

The assembly produced 616 genes of *B. aphidicola* with a GC content of 25.2% and an N_50_ of 1′206 bases. Of these genes, 569 could be annotated with GO-terms. In comparison, *B. aphidicola* of *A. glycines* has 618 genes. Our assembly reached a *Proteobacteria* BUSCO score of 73.3% complete genes (Supplementary Table 3). Such a low score was expected due to the reduced genome of *B. aphidicola.*

We identified 1′706 *H. defensa* and APSE genes. GC content of the genes was 41.35% and 1′326 genes could be annotated with GO-terms. In comparison, *H. defensa* strain ZA17, from *A. pisum*, contains 2′370 genes. In our assembly, 92.3% of the *Proteobacteria* BUSCO genes were complete, 3.2% were fragmented (Supplementary Table 3).

### Mapping

**Fig. 2:**
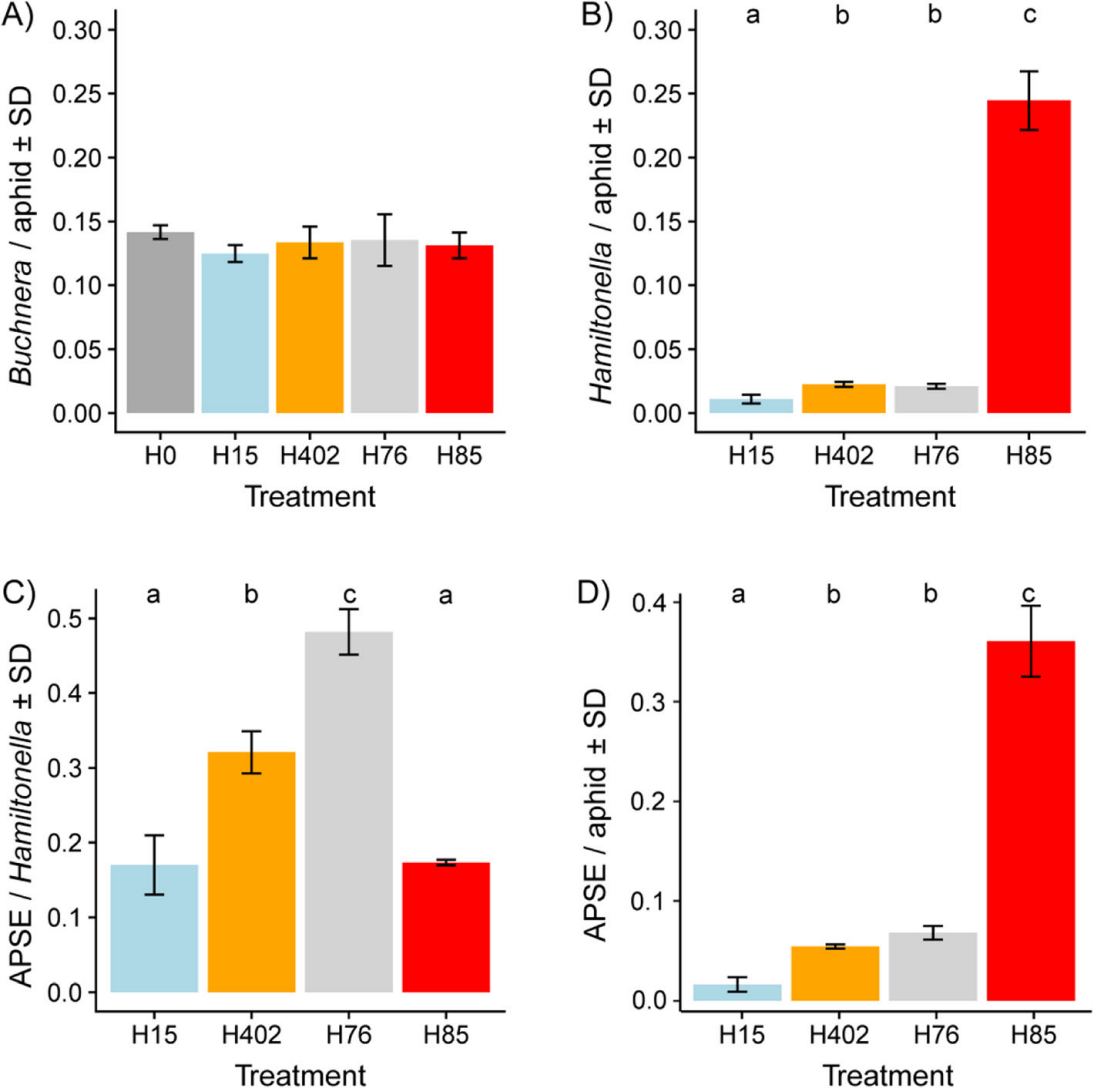
Over-replication of *H. defensa* strain H85. **A** Ratio of reads mapped to *B. aphidicola* and to aphid genes, averaged by treatment (uninfected (H0, dark grey) or *H. defensa*-infected aphid hosts (infecting strains H15 (blue), H402 (orange), H76 (light grey), H85 (red)). A one-way ANOVA comparing the effect of treatment on the read ratio was not significant (F_(4,14)_ = 0.84, *p* = 0.52). **B** Ratio of reads mapped to *H. defensa* genes and to aphid genes. A one-way ANOVA comparing the effect of treatment on the log read ratio was significant (F_(3,11)_ = 275.57, *p* < 0.001). Treatments with different letters are significantly different in pairwise post-hoc tests (Tukey’s HSD). **C** Ratio of reads mapped to APSE genes and to *H. defensa* genes. A one-way ANOVA comparing the effect of treatment on the read ratio was significant (F_(3,11)_ = 109.77, *p* < 0.001). Treatments with different letters are significantly different in pairwise post-hoc tests (Tukey’s HSD). **D** Ratio of reads mapped to APSE genes and to aphid genes. A one-way ANOVA comparing the effect of treatment on the read ratio was significant (F_(3,11)_ = 260.63, p < 0.001). Treatments with different letters are significantly different in pairwise post-hoc tests (Tukey’s HSD)

Over all 19 libraries included in the analysis, 73% of read pairs could be mapped (Supplementary Table 1). Across all libraries, the majority of read pairs (61%) mapped to aphid genes. Approximately 8% of reads mapped to *B. aphidicola*, and the ratio of *B. aphidicola* to aphid reads was stable across treatments (Fig. 2 A). In contrast, the percentage of reads mapped to *H. defensa* was highly variable. It amounted to 12.7% in aphids infected with *H. defensa* H85, which is much higher than in aphids infected with H76 and H402 (1.4 and 1.5%, respectively) or H15 (0.6%). Accordingly, the ratio of *H. defensa* to aphid reads varied significantly among treatments (Fig. 2 B). Notably, the APSE to *H. defensa* read pair ratio was highest in H76, intermediate in H402 and lowest in H15 and H85 (Fig. 2 C). The APSE to aphid read pair ratio was highest in aphids infected with H85, which was a consequence of the higher abundance of this strain and not of a higher APSE expression (Fig. 2 C and D).

### Differential gene expression in aphids

**Fig. 3:**
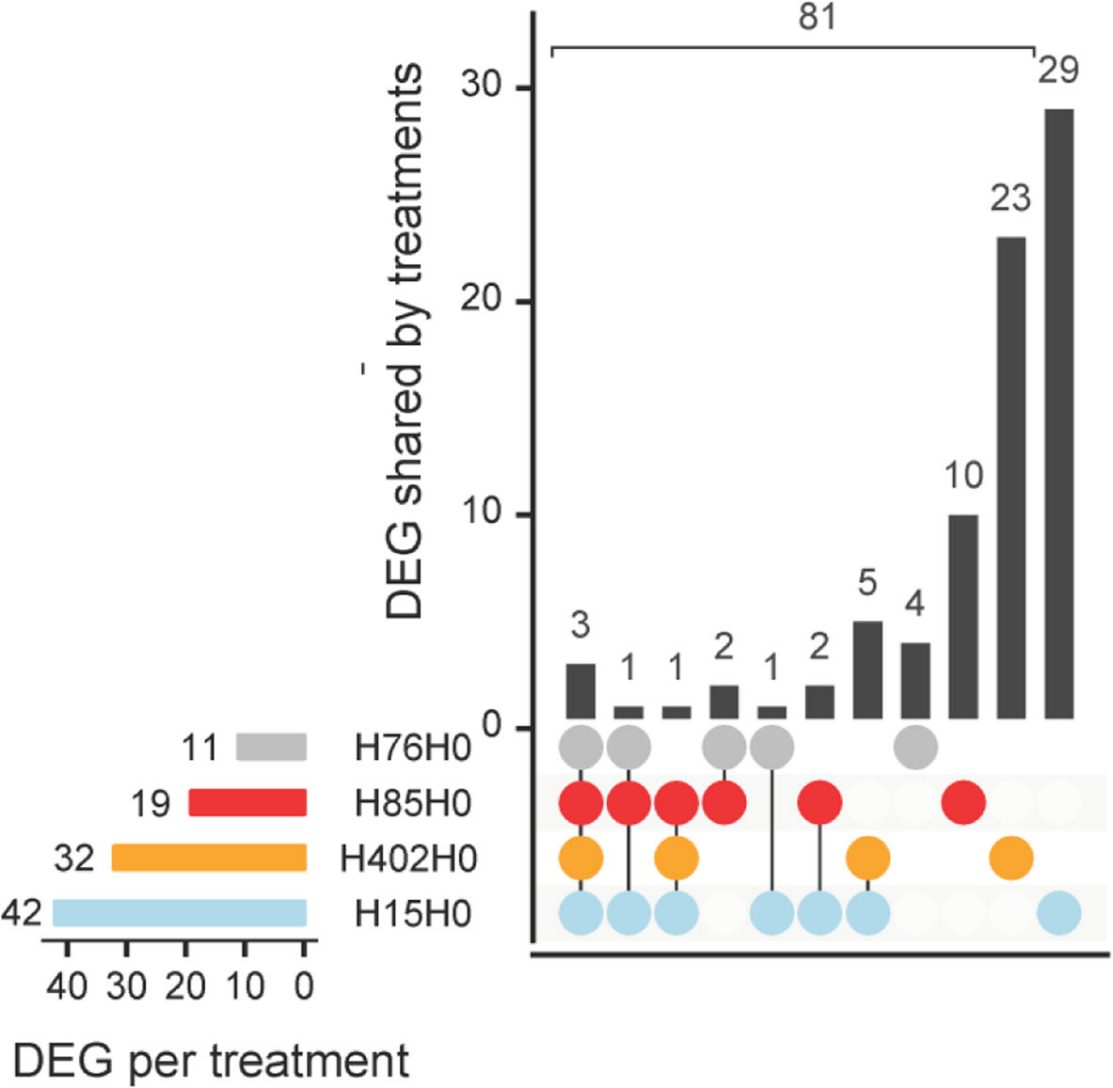
Few differentially expressed aphid genes between treatments. The horizontal bars indicate the total number of differentially expressed genes (DEG) per treatment. Vertical bars indicate which genes are differentially expressed in all four treatments (leftmost column), in two or three treatments (middle columns) or in only one treatment (rightmost four columns). The sum of all vertical bars corresponds to the total number of affected genes over all four treatments

Gene expression of aphids infected by each of the four *H. defensa* strains was individually compared to gene expression of uninfected aphids (H0). There were between 11 and 42 differentially expressed genes (DEG) (Fig. 3, Supplementary Table 4). Out of the 81 aphid genes affected by the presence of *H. defensa*, only three were differentially expressed in the presence of all four *H. defensa* strains: G patch domain-containing protein 11, an uncharacterized protein and peptide chain release factor 1 (Fig. 3 and Supplementary Table 5).

**Fig. 4:**
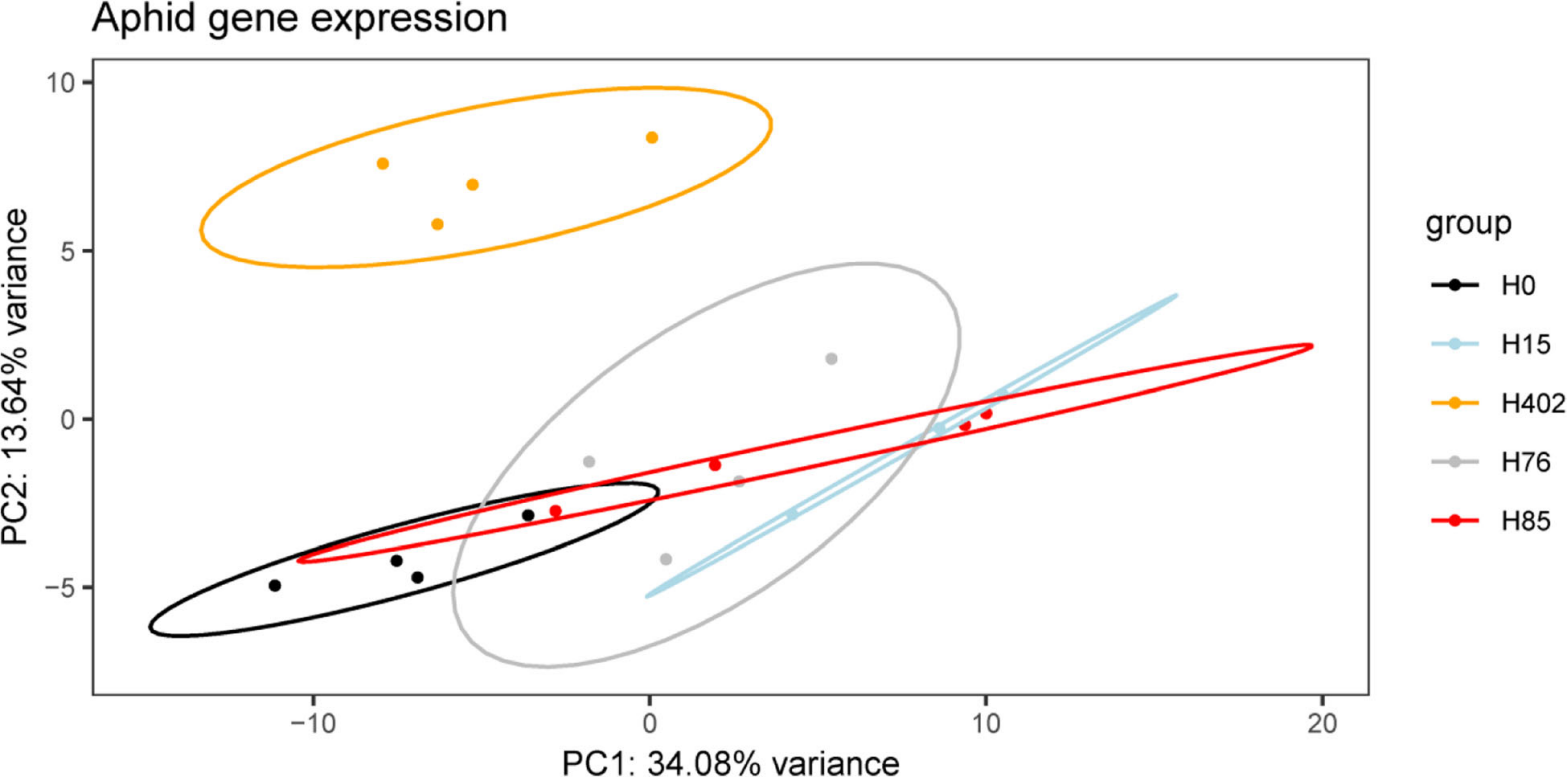
Aphid gene expression changes most upon infection with *H. defensa *strain H402. PCA of the normalised and variance stabilisation transformed read count of all aphid genes expressed in uninfected (H0, black) and *H. defensa *infected aphids (infecting strains: H15 (blue), H402 (orange), H76 (grey), H85 (red))

The most prominent changes to gene expression were observed between aphids infected with H402 and uninfected aphids. In a PCA of aphid gene expression patterns, libraries of treatment H402 were clearly separated from other treatments (Fig. 4), and the median log2 fold change of the 32 DEG between H402 and H0 was higher than when aphids were infected by other *H. defensa* strains (Supplementary Table 4). The function of 25 of the 32 DEG could not be determined; blasting against nucleotide and protein databases only yielded references to uncharacterized proteins. Of these 25 unknown genes, 18 were only differentially expressed in presence of H402 (Supplementary Table 5). Libraries of treatments other than H402 clustered closer to the control treatment H0, which was also reflected in lower median fold changes (Supplementary Table 4). Aphids infected with H15 differentially expressed 42 genes compared to H0, aphids infected with H85 differentially expressed 19 genes and aphids infected with H76 differentially expressed 11 genes compared to H0 (for a complete list of differentially expressed genes see Supplementary Table 5). We found no enriched GO-terms within these differentially expressed sets, regardless of whether we analysed DEG of each treatment or DEG shared between different treatments.

**Fig. 5:**
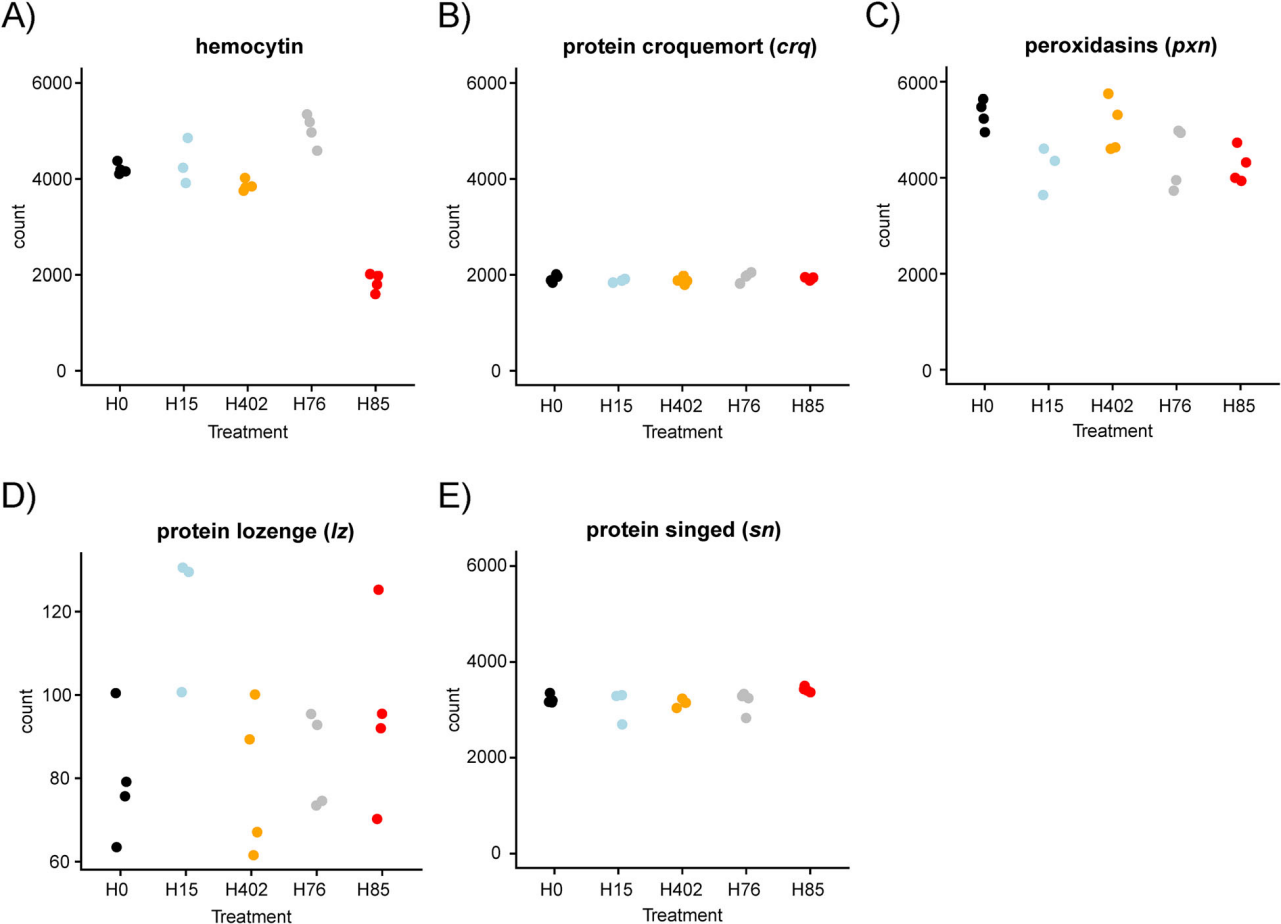
Hemocyte marker downregulated in presence of *H. defensa* strain H85. Normalised read counts of **A**) hemocytin and **B**) protein croquemort. For **C**) read counts of two transcripts annotated with “peroxidasin” and “Low quality protein: peroxidasin” were combined. Normalized read counts of D) lozenge and E) protein singed. Aphids were either infected by *H. defensa* (strains H15 (blue), H402 (orange), H76 (grey) or H85 (red)) or uninfected (H0, black)

To investigate the difference in aphid phenotype caused by the genotypically similar *H. defensa* strains H15 and H85, we also compared aphids infected by H85 to aphids infected by H15, identifying six differentially expressed genes (protein aubergine, nuclear pore complex protein Nup50, ubiquitin-related modifier 1, hemocytin and two uncharacterized proteins. See Supplementary Table 6). Comparison with the other treatments showed that aphids infected by H85 expressed less hemocytin than aphids infected by H15 as well as aphids infected by H76 or H402 and uninfected aphids (Fig. 5 A). The homolog of hemocytin in *Drosophila melanogaster* is known as hemolectin (*hml*), and genes of the *hml* family are markers of hemocytes [46]. However, other *Drosophila* hemocyte markers detected in our gene expression data – croquemort (*crq*), protein singed (*sn*), protein lozenge (*lz*) and two transcripts annotated as peroxidasin (*pxn*) [46–50] – were not significantly differentially expressed in presence of *H. defensa* (Fig. 5 B-E). Protein aubergine was upregulated in aphids infected with H85 (log2 fold change = 1.09, adjusted *p*-value< 0.001) but also in aphid infected with H76 (log2 fold change = 0.6 adjusted p-value< 0.001) compared to uninfected aphids (Supplementary Table 5). Finally, ubiquitin-related modifier 1 was significantly downregulated in aphids infected with H15 (Supplementary Table 5).

### Differential gene expression between *Hamiltonella defensa* strains

**Fig. 6:**
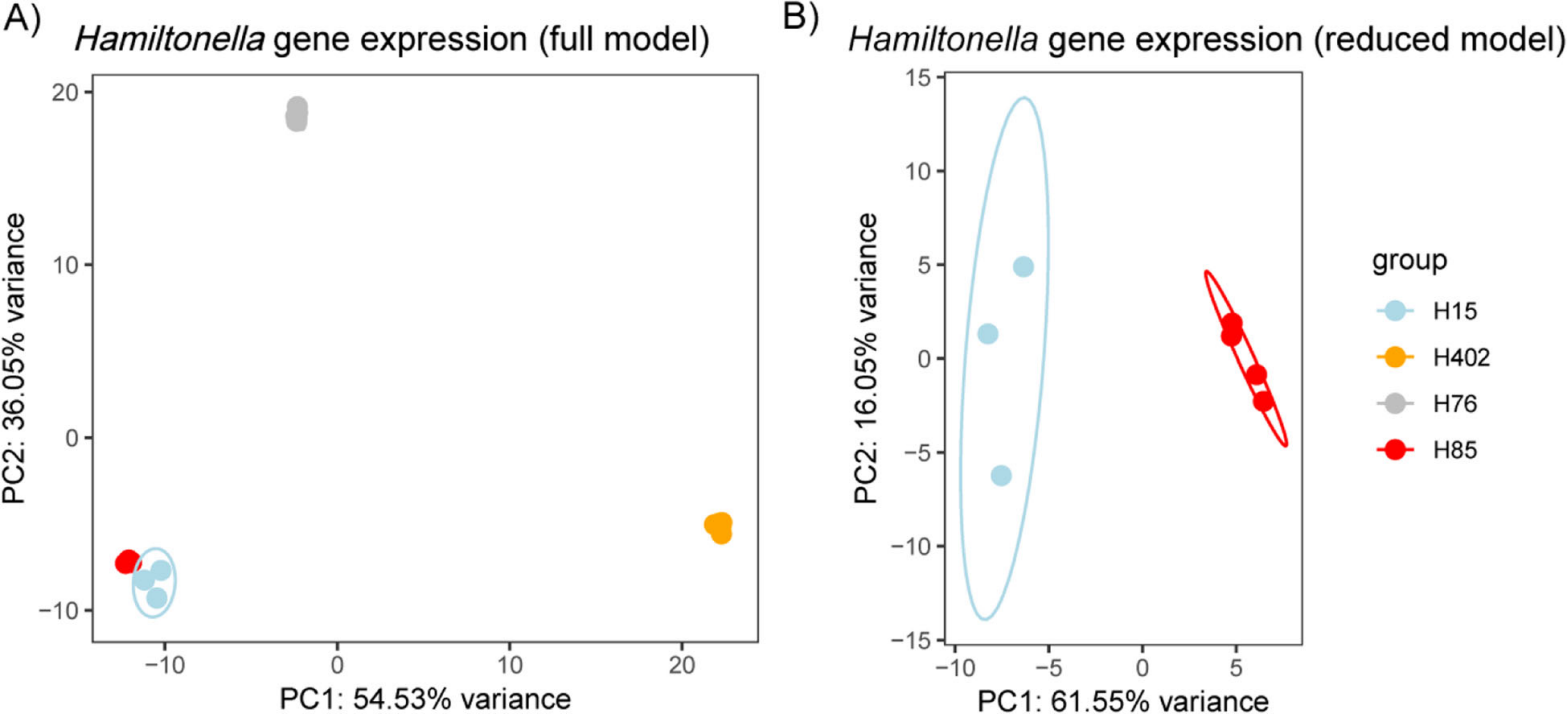
Gene expression of the four *H. defensa* strains is very different. PCA of the normalised and variance stabilisation transformed read count of all genes expressed *H. defensa* (H15 (blue), H402 (orange), H76 (grey), H85 (red)). **A** Full model containing all libraries except H15R1. **B** Reduced model containing only libraries from treatment H15 and H85.The 95% confidence ellipse is sometimes covered by the dots indicating the samples’ location in the PCA plot

For all analyses, gene expression of *H. defensa* and their APSE bacteriophage was combined and will be referred to as “*H. defensa* gene expression”. A PCA of *H. defensa* gene expression patterns segregated H76 and H402 distinctly from H15 and H85 (Fig. 6 A). As with aphid expression, we conducted a separate analysis comparing just H15 and H85; this showed a clear distinction in gene expression patterns between these two strains as well (Fig. 6 B).

To assess differences between the four *H. defensa* strains, we used the costly H85 as a reference. In the full model, H15 differentially expressed only 60 (or 4.1%) of 1′477 *H. defensa* genes that were included in the analysis compared to H85, but H402 and H76 differentially expressed 669 and 578 (or 46 and 39%) of all genes. In the DEG between different *H. defensa* strains, seven GO-terms were significantly enriched (Table 1): ‘Pathogenesis’ in the DEG between H402 and H85, and GO-terms linked to translation (‘structural constituent of ribosome’, ‘ribosome’, ‘rRNA binding’ and ‘translation’) in the DEG between H15 and H85.

**Table 1:** Differentially expressed Gene Ontology terms in *H. defensa*

DEG List	GO-term	GO Category	p-value	FDR value
H15 vs H85	structural constituent of ribosome	Molecular function	3.99E-12	7.09E-09
	ribosome	Cellular component	1.02E-11	7.09E-09
	rRNA binding	Molecular function	3.40E-09	9.01E-07
	translation	Biological process	9.35E-08	2.07E-05
H76 vs H85	host cell membrane	Cellular component	1.40E-04	0.07
H402 vs H85	pathogenesis	Biological process	9.79E-06	0.02
Shared DEG H76 vs H85 H402 vs H85	interspecies interaction between organisms	Biological process	4.45E-06	0.01

Lists of differentially expressed *H. defensa* genes were tested for GO-term enrichment using Blast2Go’s Enrichment Analysis pipeline. Lists of GO-terms were reduced to the most specific terms. GO-category, p-value and false discovery rate (FDR) are indicated for each term

**Fig. 7:**
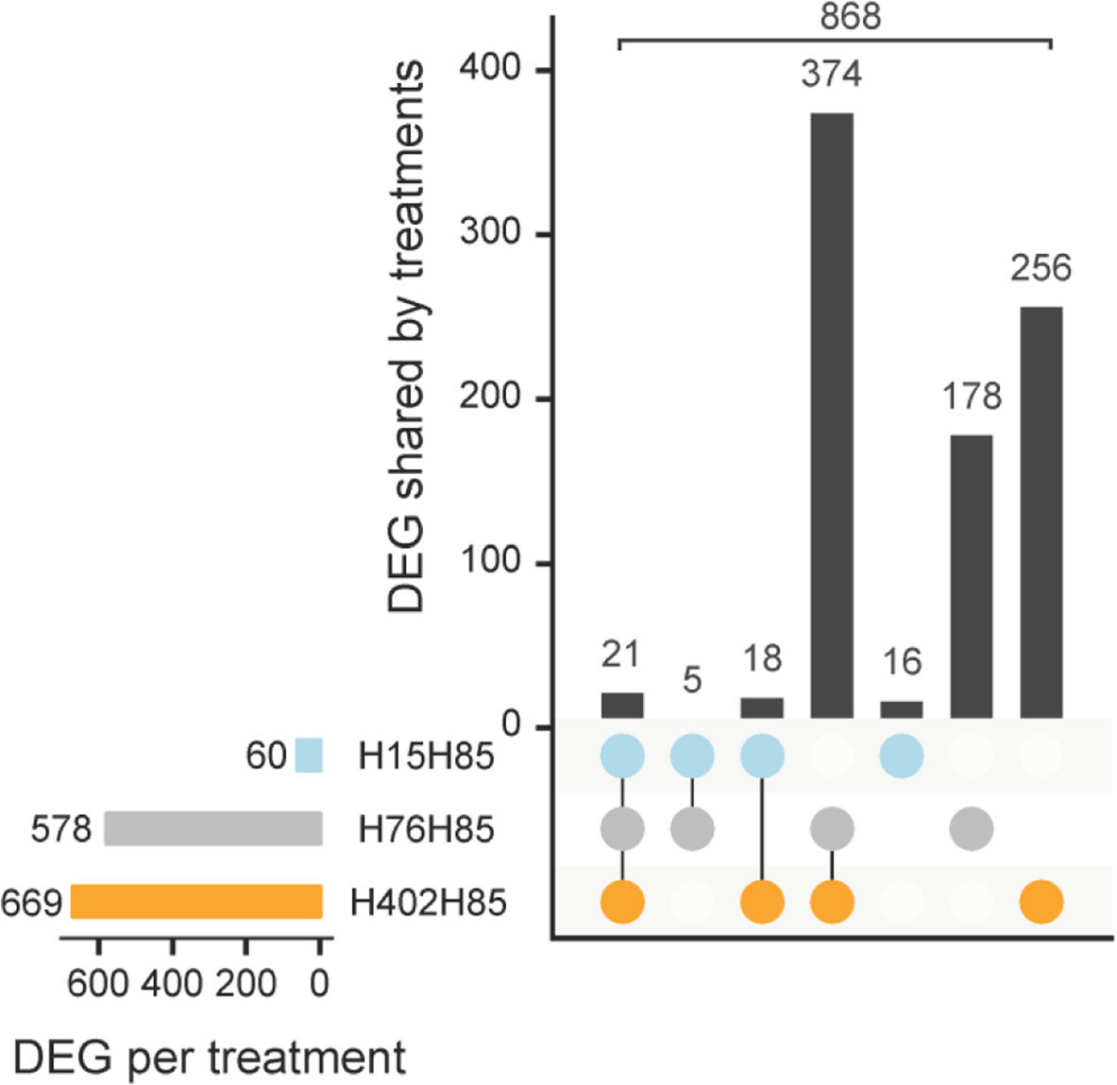
Differentially expressed *H. defensa* genes shared between the strains, relative to H85. Gene expression of *H. defensa* strains H15 (blue), H402 (orange) and H76 (grey) in comparison to strain H85. The horizontal bars indicate the total number of differentially expressed genes (DEG) per treatment. Vertical bars indicate which genes are differentially expressed in all three treatments (leftmost column), in three or two treatments (middle columns) or in only one treatment (rightmost three columns). Data for all comparisons are from the full model with all strains. The sum of all vertical bars corresponds to the total number of affected genes over all four treatments

A total of 21 genes were differentially regulated in all of the pairwise comparisons between strains H15, H76, H402 and H85 (Fig. 7, Supplementary Table 7). These genes were not significantly enriched for any GO-terms. Strains H76 and H402 shared more than half of the genes that they differentially expressed compared to H85: 64.7 and 55.9%, respectively. The 374 shared DEG were significantly enriched for the GO-term ‘interspecies interaction between organisms’ (Table 1). Among the 25 genes annotated with ‘interspecies interaction’, 12 genes also belonged to the GO-term ‘viral entry into host cells’.

Apart from YD-repeat toxin (in H76, H15 and H85) and CdtB toxin (in H402), we identified 31 APSE genes that were expressed in all strains. All 31 APSE genes were upregulated in H76 compared to H85, while 18 were upregulated in H402 compared to H85. Between H15 and H85 no APSE genes were differentially expressed (Supplementary Table 7). The YD-repeat toxin of H15 was identical to the toxin already known from H85. Finally, a total of 29 ribosomal proteins were differentially expressed in one or several *H. defensa* strains compared to H85 (Supplementary Table 7). Apart from the 50S ribosomal protein L34, which was expressed at significantly lower levels in H76 than H85, expression of ribosomal proteins in H85 was generally equal or lower than in other strains.

### Differential gene expression in *B. aphidicola*

Based on previous studies, changes in gene expression of the obligate endosymbiont *B. aphidicola* were expected to be subtle [51]. Indeed, of the 553 genes included in the analysis after removal of genes with low expression, only three were differentially expressed when the host was infected with *H. defensa.* One gene, a signal peptidase II showed strong variation between replicates of the same treatments, leading to exclusion from analysis. The two other genes, the tRNA-threonylcarbamoyltransferase complex dimerization subunit type 1 *TsaB* and the DNA-binding transcriptional regulator *Fis* were both downregulated in presence of *H. defensa* H85 (Supplementary Table 8).

### Correlation of aphid and secondary endosymbiont gene expression

To correlate gene expression between different organisms, we followed the two approaches described in Smith et al. [51]. First, we used the correlation approach [51], for which invariant *H. defensa* and aphid genes were removed from the data (Table 2). The regularized log-transformed read counts of 1′242 *H. defensa* genes and 1′288 aphid genes (Table 2) were correlated to each other in all possible pairwise combinations. This led to the identification of clusters of aphid genes that correlated – across all libraries of treatments H15, H76, H85 and H402 – with the same *H. defensa* genes, and vice versa. These clusters of aphid and *H. defensa* genes will be called ‘aphid modules’ or ‘*H. defensa* modules’ hereafter. The eigengenes – the first principal component of the expression matrix of the corresponding module – of the 11 aphid and 13 *H. defensa* modules were correlated to detect instances where the two species might influence each other’s gene expression. Note that modules were labelled with names indicating which species’ gene expression was compared (‘ApHdef’ for the comparison between aphid and *H. defensa*) and whether the module consists of aphid genes (A1-A11) or *H. defensa* genes (H1-H13).

**Table 2:**
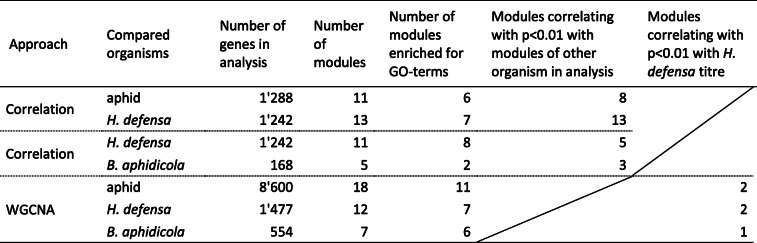
Modules of co-expressed genes correlate with each other and with *H. defensa* titre

Genes were clustered according to their expression patterns across all libraries containing *H. defensa* (except the heavily contaminated library H15R1) using two approaches, a correlation approach as described in Smith et al. [51] and weighted correlation network analysis (WGNA). The genes used for analysis were clustered into modules of co-expressed genes. These modules were tested for GO-term enrichment, for correlation with modules of another organism and for correlation with *H. defensa* titre

The correlation approach identified two aphid modules that contained genes identified as interesting during the differential expression analysis. One of them was the aphid module ApHdef-A3, in which GO-term ‘ligase activity’ was enriched (Supplementary Table 9 B). Among the 22 genes in this module was hemocytin, a gene that was shown to be strongly downregulated in the presence of H85 by the differential gene expression analysis. The genes in ApHdef-A3 might be influenced in their expression by the genes in the *H. defensa* module ApHdef-H10, since the eigengene of the aphid module ApHdef-A3 showed a strong negative correlation with the eigengene of the *H. defensa* module ApHdef-H10 (r(13) = − 0.90, *p* < 0.001) (Supplementary Fig. 2, Supplementary Table 10 B). In the *H. defensa* module ApHdef-H10, no GO-terms were enriched, but the module contained the gene AS3p2_hypothetical_protein_CDS_BJP42_RS11500. This gene was found to be strongly upregulated in *H. defensa* H85 compared to all other strains in the differential gene expression analysis (Log2 fold change in H15 = -2.13, in H402 = -3.01 and in H76 = -5.69 compared to H85).

Of further interest was the aphid module ApHdef-A2, which contained – among its 141 genes – 20 of the 25 genes encoding uncharacterized aphid proteins that were strongly differentially expressed in presence of H402. The eigengene of the aphid module ApHdef-A2 correlated well with three *H. defensa* modules: ApHdef-H7 (r(13) = 0.77, *p* < 0.001) which was enriched for GO-terms associated to ATP synthesis, ApHdef-H12 (r(13) = − 0.82, p < 0.001) without associated GO-terms and ApHdef-H13 r(13) = − 0.81, *p* < 0.001) which was enriched for GO-terms such as ‘integral component of membrane’ and ‘outer membrane’ (Supplementary Fig. 2 and Supplementary Table 10 B).

Several additional aphid and *H. defensa* modules were conspicuous as they correlated very strongly with each other. For example, there was a strong negative correlation between the eigengene of the aphid module ApHdef-A1 and the eigengenes of the *H. defensa* modules ApHdef-H5 (r(13) = − 0.9, *p* < 0.001, Supplementary Fig. 2) and ApHdef-H8 (r(13) = − 0.89, p < 0.001, Supplementary Fig. 2). Of the three modules, only module ApHdef-H8 was associated with GO-terms (‘mismatch repair complex’, ‘outer membrane’, ‘DNA binding’). Finally, there was strong correlation between the eigengene of the aphid module ApHdef-A4, which was enriched for GO-terms related to protein folding and gene expression, and the eigengenes of two *H. defensa* modules: module ApHdef-H11 (r(13) = − 0.91, *p* < 0.001), in which no GO-terms were enriched, and module ApHdef-H6 (r(13) = 0.92, p < 0.001), in which the terms ‘modification of morphology or physiology of other organism involved in symbiotic interaction’, ‘dicarboxylic acid biosynthesis process’ and ‘RNA-dependent DNA biosynthetic process/polymerase activity’ were enriched (Supplementary Fig. 2 and Supplementary Table 10 B). Notably, the *H. defensa* module ApHdef-H6 contained genes that were more or mainly expressed by strain H402, among these also the APSE gene that encodes the CdtB-toxin. The eigengene of the aphid gene module ApHdef-A5, which contained the differentially regulated ubiquitin-related modifier *urm1* was not strongly correlated with eigengenes of any *H. defensa* gene modules (Supplementary Fig. 2).

In a second approach, we used weighted gene correlation network analysis (WGCNA) to identify modules of aphid or *H. defensa* genes that correlated to the *H. defensa* to aphid read ratio – an approximation of *H. defensa* titre – of each replicate (Table 2). The approach clustered aphid genes into 18 modules and *H. defensa* genes into 12 modules.

We identified two aphid modules whose eigengenes correlated significantly positively with *H. defensa* titre: Aphid-w9 (r(13) = 0.69, *p* = 0.005) and Aphid-w10 (r(13) = 0.64, *p* < 0.001) (Supplementary Table 9 A). While no GO-terms were enriched in Aphid-w9, Aphid-w10 was associated with the GO-term ‘actin nucleation’. The WGCNA-approach also identified two *H. defensa* modules whose eigengenes correlated significantly with titre: Hdef-w11 (r(13) = 0.81, p < 0.001), in which no GO-terms were enriched, and Hdef-w8 (r(13) = 0.77, *p* < 0.001), in which the GO-term ‘type II secretion system (T2SS) complex’ was enriched. Targeted inspection of the expression of the T2SS genes showed, however, that this result was based on two T2SS-genes, *gspE* and *gspF*. Other T2SS genes, such as *gspD, gspL* and *gspM* were assigned to modules that did not correlate with titre. During the investigation we found that several genes of the T2SS, that were previously found in *H. defensa* of pea aphids [52], were not assembled from our sequencing data. Notably, H76 only expressed one out of five T2SS genes, *gspD*.

### Correlation of primary and secondary endosymbiont gene expression

The same two correlation approaches as described above were applied to *Buchnera aphidicola* and *H. defensa* genes (Table 2). The strongest correlations were found between the eigengene of the *B. aphidicola* module BapHdef-B4 (no enriched GO-terms or KEGG pathways) and the eigengenes of the two *H. defensa* modules BapHdef-H5 (r(13) = 0.85, *p* < 0.001), which contained the APSE gene encoding the CdtB-toxin and in which GO-terms such as ‘viral life cycle’ and ‘interaction with host’ were enriched, and BapHdef-H9 (r(13) = − 0.85, p < 0.001), in which the GO-term ‘macromolecule transmembrane transporter activity’ was enriched (Supplementary Fig. 3 and Supplementary Table 11 B).

The WGCNA approach identified one module of *B. aphidicola* genes, Bap-w6, whose eigengene’s expression correlated negatively (r(13) = − 0.78, *p* = 0.001) with *H. defensa* titre (Supplementary Table 11 A). No KEGG pathways or GO-terms were enriched in Bap-w6, but the module contained the DEG tRNA-threonylcarbamoyltransferase complex dimerization subunit type 1 *TsaB* of *B. aphidicola*.

### Characterisation of *Hamiltonella defensa* strains

**Fig. 8:**
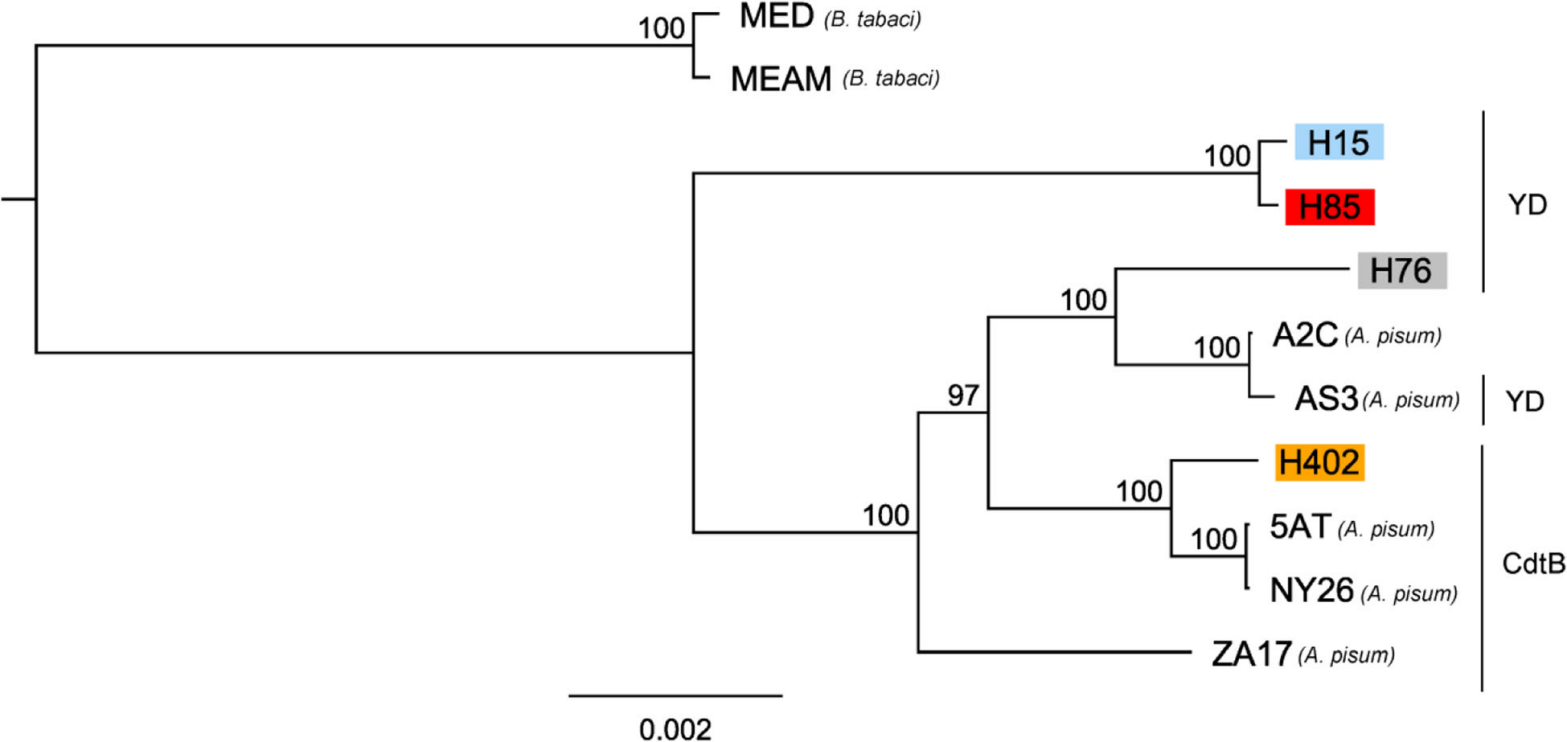
*H. defensa* from different aphid species but with similar APSE toxin cluster together. Phylogram based on 161 shared and complete BUSCO genes extracted from *H. defensa* strains of *A. fabae* (H15, H76, H85, H402), *Bemisia tabaci* (MED, MEAM) and *Acyrthosiphon pisum* (A2C, AS3, NY26, ZA17). Nodes are labelled with branch support based on percentage of bootstrap replicates that recovered the same node, and the toxin that the APSE encodes is indicated on the right (A2C has no APSE). Branch length is proportional to scale bar (unit: amino acid substitutions per site)

To place our *H. defensa* strains in a phylogeny with other sequenced strains, 161 BUSCO genes were extracted from our transcriptome data and from publicly available *H. defensa* genomes. Strain MED from *Bemisia tabaci* was used as an outgroup during phylogeny construction (Fig. 8). Strains H15 and H85 were closely related and formed a separate clade that was well supported and basal to the other aphid-infecting strains we included. Strain H76 clustered with *H. defensa* A2C and AS3 from *A. pisum*, while strain H402 clustered with NY26 and 5AT from *A. pisum*.

The APSE toxin cassettes of strains H76, H85 and H402 had already been sequenced [43]. The toxins assembled from the RNA-Seq data in this experiment confirmed our expectations from that prior sequencing: Strain H85 carried a YD-repeat toxin that was identical to the reference toxin from H85 (NCBI GenBank: MW535750.1). H15 carried the same toxin as H85. The YD-repeat toxin of H76 agreed with our expectations from the reference gene (NCBI GenBank: KU175898.1) but was longer and completed by a stop-codon. The CdtB toxin of H402 was retrieved from our data with one missense substitution (Glycine-> Valine) compared to the reference gene (NCBI GenBank: KU175897.1).

## Discussion

We used a triple RNA-Seq approach to monitor gene expression of the host *A. fabae* and its primary endosymbiont *B. aphidicola* in presence or absence of the secondary endosymbiont *H. defensa*. The four *H. defensa* strains used in the experiment show large variation in their gene expression and affect the aphid host’s gene expression in different ways.

### Host and *H. defensa* influence each other

The triple RNA-Seq approach was a necessity, as neither *B. aphidicola* nor *H. defensa* of *A. fabae* can currently be cultured, and advantageous as it allows investigating the interaction between the three different species through simultaneous analysis of gene expression in host and endosymbionts. Aphids and *H. defensa* seem to interact strongly with each other: The majority of *H. defensa* and aphid gene expression modules showed significant correlations with modules of the other species, and in four instances absolute correlation values were 0.9 or higher. Of immediate interest in further research will be the *H. defensa* gene module that showed a strong negative correlation with the hemocytin-containing aphid module. The genes contained in this *H. defensa* module might suppress the host’s hemocytin-expression. A candidate for suppression of hemocytin-expression is the *H. defensa* gene AS3p2_hypothetical_protein_CDS_BJP42_RS11500, which is encoded on a plasmid in *H. defensa* strain AS3 of pea aphids. The gene is strongly overexpressed in H85 only, and it is the DEG with the highest log2 fold change between the closely related strains H15 and H85.

### *H. defensa* titre does not fully explain the impact on host gene expression

The high titre of H85 has curiously little impact on aphid gene expression: The host’s gene expression reacted more strongly to presence of the intermediate-density H402 than to H85. Additionally, only two aphid gene modules correlated with *H. defensa* titre, indicating that titre alone can only explain few expression changes in groups of co-expressed aphid genes. This is further supported by the fact that the two closely related strains H15 and H85, even though they differ strongly in titre (see Kaech et al. [44] for qPCR estimates of *H. defensa* density) and in the costs they impose on their host, induced significant expression differences in six genes only, three of which we will discuss in further depth below.

### Deregulation of protein aubergine

In *Drosophila,* protein aubergine (*aub*) is part of the piRNA pathway [53]. In our experiment, *aub* was upregulated in presence of H85 and to a lesser extent in presence of H76. Other genes related to the piRNA pathway, such as protein argonaute 3 and Piwi-like protein Siwi, were expressed but not differentially regulated*.* Due to the piRNA pathway’s importance to reproductive cells, deregulation could be related to altered fertility. Yet, *aub* was upregulated both in presence of the *H. defensa* strain H85, which strongly reduces the host’s fertility, and strain H76 with no detectable impact on host fertility.

### Reduced *urm1* expression in presence of strain H15

In presence of *H. defensa* H15, expression of the aphid gene ubiquitin-related modifier 1 (*urm1*) was downregulated compared to uninfected aphids and aphids infected by other *H. defensa* strains. In yeast, *urm1* deficiency leads to failure to decrease the expression of the amino acid permease GAP1 – which is used for uptake of low-quality nitrogen-sources – even though high-quality nitrogen-sources are available in the environment [54]. Assuming that there are similar mechanisms in the aphid, decreased *urm1* expression might alter which nitrogen-sources are used by the aphid and which are available to *H. defensa*. Thus, the decreased *urm1* expression in aphids infected with H15 might lead to metabolic control over *H. defensa* H15, which could prevent H15 from over-replicating like its close relative H85. However, we did not find any amino acid permeases that were differentially expressed or other indication for altered nitrogen uptake in aphids with reduced *urm1* expression. Additionally, the aphid gene module containing *urm1* did not correlate strongly with any *H. defensa* modules. Thus, there were no robust ties linking *urm1* downregulation to changes in *H. defensa* gene expression or a potential titre control mechanism.

In *Drosophila*, *urm1-*deficiency was also linked to increased cytoprotective JNK-signalling [55]. If reduced *urm1-*expression had a similar impact on JNK-signalling in aphids, it would be perceivable that this could affect the titre of the bacterial symbiont *H. defensa,* as the JNK pathway is involved in defence against bacterial pathogens in pea aphids [56]. Although we identified several genes associated with the JNK-pathway in our assembly (peroxidasin 1, transcription factor kayak, transcription factor AP-1, oxidation resistance protein 1, catalase), they were not differentially expressed in presence of any *H. defensa* strain. Thus the effect of reduced *urm1*-expression in aphids infected with H15 remains unclear.

### Strain H85 co-occurs with downregulation of hemocytin

Presence of *H. defensa* H85 co-occurred with differential expression of the aphid gene hemocytin. Hemocytin was first described in *Bombyx* [57] and its homolog in *Drosophila* is called hemolectin (*hml*). Genes of the *hml* family include domains that are typically observed in vertebrate and arthropod clotting factors. They are mainly associated with coagulation after wounding [58, 59], together with fondue and phenoloxidases [60]*.* In *A. pisum* phenoloxidases are downregulated in presence of *H. defensa* [61], but in our experiment, the one aphid phenoloxidase assembled was not differentially expressed in presence of any *H. defensa* strain. A homolog of fondue was not assembled.

It has been noted that in adult *Drosophila,* infection with Gram-negative bacteria leads to increased *hml* expression [62]. However, our *A. fabae* clone did not significantly upregulate hemocytin expression in response to infection with *H. defensa* strains H15, H76 or H402, and when infected with strain H85, the clone actually experienced strong downregulation of hemocytin. Such a downregulation has also been observed in *A. pisum* infected with *Regiella insecticola,* but not in *A. pisum* infected with *H. defensa* [63]*.* In a gene expression study from *A. pisum*, hemocytin expression seemed quite specific to hemocytes (see Supplementary Table S4 in [64]), which suggests that the gene might – similar as *hml* in *Drosophila* [46] – be strongly expressed by hemocytes. We thus first wondered whether H85 decreased the number of aphid hemocytes. Yet, the observed decrease in hemocytin expression in presence of H85 is unlikely related to reduced numbers of hemocytes, as the expression of other hemocyte markers known from *Drosophila* did not significantly change. Instead, H85 might have specifically inhibited expression of hemocytin.

Overall, our results motivate further studies investigating whether altered hemocytin expression in *A. fabae* is truly not related to altered hemocytes numbers, and whether there might be a causal connection of a reduced hemocytin expression to over-replication of the secondary endosymbiont H85.

### Strain H402 activates a cluster of unknown aphid genes

Another factor determining the host’s reaction to a *H. defensa* strain may be the APSE it contains: In general, the YD-repeat-toxin encoding strains H15, H76 and H85 elicited less pronounced differential expression in the aphid host than the CdtB-toxin encoding H402. Presence of H402 was connected to strong differential expression (mostly upregulation) of 25 aphid genes with unknown function, 18 of which were only differentially expressed in presence of H402. Even though the aphid gene module containing these genes was linked to three *H. defensa* gene modules, in which GO-terms related to membrane composition and ATP synthesis were enriched, the mechanism behind the impact of strain H402 on these aphid genes of unknown function remains unclear. Nevertheless, our experiment points towards a clear difference in the aphid’s reaction to the CdtB-toxin encoding strain H402 compared to all other strains.

### *B. aphidicola* shows little reaction to presence of *H. defensa*

Even though *H. defensa* is known to require essential amino acids generated by the aphid’s obligate endosymbiont *B. aphidicola* for survival [65], *B. aphidicola* showed little reaction to the presence of *H. defensa*, both in terms of differential gene expression and density. The few *B. aphidicola* genes that were significantly differently expressed had low fold changes, and correlation between *B. aphidicola* and *H. defensa* gene modules was rarer and less strong than between aphid and *H. defensa* gene modules. These results are in agreement with previous studies and may not only reflect the sheltered intracellular lifestyle of *B. aphidicola* [2] but also the reduced potential of *B. aphidicola* to alter its gene expression due to the loss of regulatory genes [66, 67]. Given our data, *B. aphidicola* did not increase expression of genes involved in production of essential amino acids in presence of *H. defensa.*

### APSE activity can explain some but not all differences between *H. defensa* strains

Based on the analysis of BUSCO genes, the two *H. defensa* strains H76 and H402 were closely related to strains infecting pea aphids with same-type APSE-toxins, while strains H15 and H85 were different from any of the sequenced strains from *A. pisum*. Our phylogeny is in agreement with the phylogenies for *H. defensa* and APSE in Rouïl et al. [68], even though we used considerably more genes. Given the short evolutionary distance between the strains H15 and H85, their very different phenotypes – in terms and costs and density that they induce or reach in the host – are even more intriguing.

The three *H. defensa* haplotypes – haplotype 1 comprising of strain H76, haplotype 2 of H402 and haplotype 3 of H15 and H85 [42] – displayed markedly different gene expression patterns. Compared to H85, strains H76 and H402 differentially expressed genes enriched for GO-terms ‘pathogenesis’ and ‘interspecific interaction’. Notably APSE genes were more active in H76 and H402 than in H85 and H15. Since APSE is lysogenic [35, 37, 69] one could assume that higher APSE activity reduces a *H. defensa* strains’ density and thus the cost in terms of resources that the strains divert from the host. Therefore the higher APSE activity in H76 and H402 might explain why these strains are less costly than H85. Yet, APSE activity cannot explain the different densities of H15 and H85, as no APSE genes were differentially expressed between the two strains. Instead, GO-terms linked to ribosomes were enriched in the DEG between the two *H. defensa* H15 and H85, and 29 ribosomal proteins were differentially regulated between either H15, H76 or H402 and H85. There was a trend towards lower expression of ribosomal proteins in *H. defensa* strain H85 compared to other strains. Differential regulation of ribosomal proteins has been found to be associated with stress or different growth conditions in bacteria and yeast [70–73] and other taxa [74]. Given the over-replication of H85, stress could indeed be induced by limited nutrient availability. However, the pattern of differential regulation among *H. defensa* ribosomal proteins is complex and will require further studies.

## Conclusions

1. Variation in gene expression indicates differences in the mechanism underlying the cost to the host induced by different strains of *H. defensa*.
2. The over-replicating and very costly strain H85 impacts the aphid’s hemocytin expression, suggesting experimental investigation of the role of hemocytin on aphid secondary endosymbionts.
3. While there are strong correlations between aphid and *H. defensa* gene modules, which implies an interspecific interaction, presence of *H. defensa* impacts gene expression of the aphid’s obligate endosymbiont (*Buchnera aphidicola*) much less.

## Methods

### Aphid clones and secondary endosymbiont strains

This study uses a subset of the 12 sublines of the *A. fabae* clone A06–407 described by Cayetano et al. [42]. Clone A06–407 was naturally free of secondary endosymbionts and was infected with *H. defensa* by microinjection of hemolymph from other *A. fabae* clones between 2008 and 2012. In this experiment, we used four of these *H. defensa-*infected sublines (H15, H76, H85 and H402) as well as the subline without *H. defensa* (H0). Collection details of the clone A06–407 and the *H. defensa*-infected hemolymph donors are provided in Supplementary Table 12. Sublines were maintained on broad beans (*Vicia faba*) under environmental conditions ensuring clonal reproduction (16-h photoperiod at 18–20 °C). For each subline, 12 bean seedlings were infested with adult aphids. After reproduction, DNA of the adults was extracted to confirm aphid clone identity, presence and haplotype of *H. defensa,* and absence of *H. defensa* in case of subline H0. To avoid environmental maternal effects carrying over from stock cultures, the 12 colonies per subline were maintained at 18 °C for two generations. Nymphs of the third generation were reared at 22 °C for eight days until adult. The 12 plants per subline were divided into two batches (A and B) of six plants each and replicates of 18 aphids (R1 and R2 from plant batch A, R3 and R4 from plant batch B) were collected, with each plant of a batch contributing three aphids.

### RNA extraction

Aphids were crushed in 0.5 ml TRIzol (Thermo Fisher). The volume of TRIzol was adjusted to 1 ml and after 20 min at room temperature (RT), the samples were stored at -80 °C. All further procedures up to library quantification were conducted successively on the two batches A, comprising replicates R1 and R2, and B, comprising replicates R3 and R4.

Samples were thawed at RT and vigorously shaken by hand with 200 μl chloroform (PanReac AppliChem). After incubation for 10 min at RT and centrifugation at 4 °C, the aqueous supernatant was recovered and re-extracted with chloroform. RNA was pelleted by centrifugation after mixing with 500 μl ice-cold isopropanol (Merck) and incubating for 4 h at -20 °C. The pellet was air-dried at RT, washed with ice-cold 75% ethanol and absolute ethanol and re-suspended in 50 μl RNase-free water.

DNA was removed using the RNase-free DNase kit (Qiagen) and RNA was purified using the RNeasy Mini Kit (Qiagen). Cleaned RNA was eluted in 30 μl RNase-free water and RNA integrity was assessed with the RNA 6000 Nano kit (Agilent) on the Bioanalyzer 2100 (Agilent) (Supplementary Fig. 4). To deplete ribosomal RNA (rRNA) but maintain bacterial mRNA, 1 μg total RNA was processed with the riboZero Epidemiology kit (Illumina), using half of the recommended volume of reagents per reaction. Sample volume was adjusted to 180 μl and RNA was recovered with glycogen-assisted ethanol precipitation.

### Library preparation

RNA pellets were re-suspended in 9 μl Fragment, Prime, Finish mix from the TruSeq stranded mRNA library preparation kit (Illumina). RNA was fragmented at 94 °C for 95 s and 8.5 μl were processed according to the manual of the TruSeq kit, using half of the recommended reagent amounts and starting at the “Synthesize First Strand cDNA” step. Eleven PCR cycles were enough to achieve the desired library amplification. For barcoding, all 12 barcodes from the TruSeq RNA Single Indexes Set A (Illumina) and eight additional barcodes from Set B were used. Libraries were size selected with Agencourt AMPure XP beads (Beckman Coulter). The bead pellet was washed twice with 200 μl 80% ethanol, dried at RT and eluted in 15 μl Resuspension Buffer from the TruSeq kit. Average library fragment length, determined with the High Sensitivity DNA Analysis Kit (Agilent), varied between 421 and 460 bp, with exception of H15R1 (385 bp). Library concentrations were quantified on a Roche LightCycler 480 using the Universal Kapa library quantification kit (Kapa Biosystems).

### Transcriptome sequencing

A pool containing 10 nM of each library was sequenced by the Functional Genomics Center Zurich on an Illumina HiSeq 4000 (one lane, 150 bp paired-end reads). The number of usable reads per library surviving preliminary trimming and rRNA removal was recorded. For a second sequencing run, we designed a new library pool to approximately balance the number of usable reads per library. In total, two sequencing runs yielded 755.9 million read pairs.

### De novo assembly

To prepare reads for de novo assembly, sequencing adapters, potential primer mismatches and stretches of low-quality bases were removed from the reads using Trimmomatic v0.35 [75] with default settings except for a sliding window with a minimal average quality of 20, a minimal read length of 100 bp and a 6 bp headcrop. This strict trimming retained 83% of the read pairs. To prepare the reads for mapping, the minimal average quality trimming threshold was relaxed to 15, headcrop was deactivated, and the minimal read length was set to 75 bases. Relaxed trimming for mapping retained 93% of the read pairs, or an average of 34.7 million (± 2.8 SD) read pairs per library (Supplementary Table 1). Only reads surviving the trimming in pairs were used for subsequent steps. Reads prepared for de novo assembly and mapping were quality checked using FastQC v0.11.4 [76]. Kraken v0.10.5 [77] indicated significant human RNA contamination in library H15R1 and minor contamination in libraries H15R3 and H15R4. To recover *B. aphidicola* and aphid transcripts, the 17 uncontaminated libraries were assembled in Trinity v2.1.1 [78] with in silico read normalisation to a maximal coverage of 50 and requiring a minimal transcript length of 200. The assembled transcripts were clustered with CD-HIT-EST from the CD-HIT suite v4.6.5 [79] with a sequence identity threshold of 0.95, a band width of 50, and clustering to the most similar cluster (g = 1). Transcripts that did not reach a normalised expression metric of 0.5 TPM (transcripts per million transcripts) in at least one library were removed with the Trinity v2.4.0 script filter_low_expr-transcripts.pl. Ribosomal sequences were removed with Ribopicker v0.4.3 [80] and polyA-tails longer than 5 bases were trimmed with prinseq v0.20.14 [81]. The transcripts were assigned to the most likely organism of origin using blastn v2.2.30 [82, 83] against custom databases with default settings except for an E-value cutoff of 1e^− 8^ and a maximal number of HSPs (alignments) of 1. Transcripts that could not be assigned to an organism of origin were blasted against the nr database using diamond v0.9.22 [84] and an E-value cutoff of 1e^− 5^. 1′255 aphid transcripts had no blast results and were discarded from analysis. Transcripts assigned to aphids were annotated with GO-terms using OmicsBox [85]. Transcripts that blasted to more than one organism were discarded if the bitscore difference between best hits to different taxa was less than 100 or assigned to the best-scoring taxa if the bitscore difference was more than 100 using a custom script. Transcripts assigned to either aphids or *B. aphidicola* were separated and clustered to a sequence identity of 0.9 using CD-HIT-EST as described above.

To recover transcripts of *H. defensa* and its associated APSE, we performed de novo assembly for each *H. defensa* strain separately. For each assembly, four libraries containing the respective strain were combined, except for H15 where we excluded the heavily contaminated library H15R1. For each strain-specific assembly we retained transcripts blasting to *H. defensa* and its associated virus APSE using the same procedures and cutoffs as described above. We retrieved 4′850 transcripts from the four assemblies.

De novo*-*assembled bacterial transcripts represent operons and likely contain multiple genes. Since genes that lie on the same operon can be differentially expressed [86], the signal of a differentially expressed gene (DEG) may be diluted in transcript level analyses if other genes on the same operon are not differentially expressed. Additionally, inefficient transcription termination between convergent operons [86] could in silico merge functionally unrelated operons. To avoid such artefacts, differential gene expression in bacteria had to be assessed at gene instead of transcript level.

To identify *B. aphidicola* genes, annotations from the closely related reference genome of *B. aphidicola* from *Aphis glycines* (NCBI GenBank: NZ_CP009253.1, NZ_CP009254.1, NZ_CP009255.1) were transferred to the de novo assembled transcripts of *B. aphidicola*. This was achieved by aligning transcripts to the reference genome in Geneious v11.0.5 [87]. Manual correction steps included removal of ten chimeric *B. aphidicola* transcripts and inclusion of one unaligned transcript that matched the genome of *B. aphidicola* of *Uroleucon ambrosiae* (NCBI GenBank: CP002648.1). Sequence differences between reference genome and aligned transcripts were corrected manually and gene start and stop sites were adjusted where supported by reads. After removal of rRNA genes, annotated genes were exported for downstream analysis.

Identifying *H. defensa* genes needed a different approach, as only half of *H. defensa* and APSE transcripts aligned to related genomes. Instead of attempting the transfer of annotations, we predicted the genes from the strain-specific *H. defensa* transcriptomes: We ran Prokka v1.11 [88] while providing it with known *H. defensa* proteins (Supplementary Table 13). For each strain the predicted genes were clustered to a sequence identity of 0.8 using a length difference cutoff of 0.9 and deduplicated using a sequence identity of 0.99 and a length difference cutoff of 0.1 with CD-HIT-EST. The four gene sets were pooled, and annotations were manually curated so that strain-specific variants of the same genes were labelled identically. Gene variants were aligned with Geneious to detect and remove chimeras.

In RNA-Seq studies comparing the expression of several related bacterial strains, the correct choice of reference genome is crucial as phylogenetic distance can lead to false-positives in the differential expression analysis [89]. Since Kallisto cannot interpret non-ATGCU bases, use of a consensus sequence from all four strains would have resulted in the replacement of 29′074, or 2.36%, of all *H. defensa* bases with pseudo-random bases. To avoid such a high percentage of pseudo-random bases, we used the consensus sequence of strains H15 and H85 for differential expression analysis in *H. defensa*. This decreased the number of pseudo-random bases inserted by Kallisto to 514. The differential expression results for *H. defensa* are therefore most accurate for the two strains H15 and H85, while there may be some false positives due to phylogenetic distance for strains H76 and H402.

### Differential expression analysis

Using Kallisto v0.43.0 [90], reads trimmed for mapping were aligned simultaneously to annotated genes from *B. aphidicola*, consensus sequences of *H. defensa* and APSE genes, transcripts of *A. fabae,* and the coding DNA sequences (CDS) of the most frequent contaminant bacteria (Supplementary Table 14). The resulting abundance tables were split with a custom R function to allow organism-specific read normalisation during differential gene expression analysis using the package DESeq 2 v1.22.1 [91] and tximport v1.10.0 [92], which provided the read counts to DESeq 2, in R v3.3.2 [93]. For the analysis, aphid transcripts were merged to gene level. From the assembly 46′352 transcripts resulted, of which 2′590 did not blast to any known record in NCBI and were discarded from differential expression analysis as they might correspond to chimeric assembly artefacts or non-coding RNA. The remaining transcripts corresponded to 19′864 different Trinity ‘genes’ that were annotated as 10′809 different genes. It is important to note that Trinity assigns the ‘gene’ status purely based on mathematical, not biological, information. For the differential expression analysis, we therefore merged all Trinity genes that were annotated as the same gene. For this, the list of annotations was manually curated, removing differences in gene annotations such as the terms ‘Predicted’ or ‘isoform X1’.

Differential expression analysis was done separately for each species. For analysing aphid gene expression, genes with less than 1 read per million were discarded from analysis, leaving 8′614 genes. To compare differences in expression of aphid genes between two treatments in DESeq 2, we used Wald tests. The differential expression models contained the two variables *batch* (A, B) and *treatment* (H0, H15, H76, H85, H402) as fixed factors. Based on AICc values, the model without interaction of the fixed factors was used. We provided the DESeq 2 result function with two significance thresholds: adjusted *p*-value alpha < 0.01 and log fold change > 0.25. We drew pairwise comparisons between aphid gene expression in the presence of a *H. defensa* strain (H15, H76, H85 or H402) to gene expression in absence of *H. defensa* (H0). Additionally, we compared aphid gene expression in presence of strain H15 to gene expression in presence of H85 using a reduced model, in which we only included libraries of treatments H15 and H85. Differential expression analysis as described above was repeated for *B. aphidicola* genes. For *H. defensa* genes*,* gene expression of strains H15, H76 and H402 was compared to gene expression of strain H85. Low-expression genes were removed from the analyses, leaving 1′477 genes. A high number of all *H. defensa* genes was differentially expressed between both H76 and H402 and the reference strain H85. Since a basic assumption of differential expression analysis is that most genes are not differentially expressed [94], this could have corrupted the differential expression model. Thus, we used a reduced model containing only libraries of treatments H15 and H85 to confirm the DEG between strains H15 and H85. Combined, the full and reduced models found 64 DEG between H15 and H85. The majority of these, 76.6%, were reported by both models. A total of 11 genes and 4 genes were only reported by the full and by the reduced model, respectively. Based on the small differences between the two models, the full model was considered stable and its results were used for further analysis.

We used UpSetR v1.3.3 [95] to visualise the number of DEG shared between treatments and pcaExplorer v2.8.0 [96] to visualise the results of principal component analyses (PCA), which used the expression patterns of all genes of each organism to segregate the libraries, showing the ordination along the two first axes.

Aphid and endosymbiont genes were annotated with GO-terms using Blast2GO v5.2.5 [97]. For GO-terms associated with each aphid, *H. defensa* and *B. aphidicola* gene see Additional File 3. The DEG of each treatment were tested for GO-term enrichment using two-tailed Fisher exact test while filtering for FDR ≤ 0.05 using OmicsBox [85]. To prevent genes with many isoforms biasing this analysis, we randomly selected one isoform per gene for GO-term enrichment.

### Phylogenetic tree

For phylogenetic analysis of *H. defensa,* we predicted BUSCO genes from the strain-specific gene sets as well as from the CDS from known and reasonably complete *H. defensa* genomes (accession numbers in Supplementary Table 13) with BUSCO v3.0.2 [98, 99] using the *Proteobacteria *dataset as reference. Single-copy complete BUSCO genes present in all strains were extracted, translated to protein sequences and aligned with MAFFT v7.273 [100]. The per-gene alignments were trimmed using trimAl v1.2 rev59 [101] and concatenated into a superalignment with Geneious. Genes with obvious frameshifts or truncations were removed, reducing the number of shared BUSCO genes from 164 to 161. Best-fit partitioning schemes and models of evolution were selected with PartitionFinder v2.1.1 [102] using RAxML [103] and the relaxed clustering algorithm [104]. Each gene corresponded to one data block, and models of evolution were selected based on AICc. Note that we did not consider models of evolution with equal base frequencies, or base frequencies determined using maximum likelihood, or amino acid frequencies estimated from mitochondrial, chloroplast, HIV viral or influenza viral datasets. The phylogenetic tree was generated with RAxML v8.2.11 in Geneious executing 500 rapid bootstrap interferences followed by a Maximum Likelihood search on data partitioned according to the PartitionFinder-results.

### Correlation of host and symbiont expression

Aphid and *B. aphidicola* gene expression were correlated to *H. defensa* gene expression using two approaches, the weighted correlation network analysis approach (WGCNA) [105, 106] and the correlation approach described in Smith et al. [51] (see module membership of aphid, *H. defensa* and *B. aphidicola* genes in Additional File 4). For the WGCNA approach, genes with a read count less than 1 read per million were discarded and read counts were log-transformed using the regularized log transformation (rlog). We followed the procedures described in Smith et al. [51] except for using signed hybrid networks and the biweight midcorrelation as a robust alternative to Pearson correlation. Briefly, modules of co-expressed genes were constructed from the rlog-transformed expression data using hierarchical clustering. Module eigengenes (defined as the first principal component of a module) were calculated and correlated with *H. defensa* titre (approximated by the *H. defensa* to aphid read ratio). After filtering out genes with low expression, the WGCA-approach included 8′600 aphid genes and 1′477 *H. defensa* genes.

For the correlation approach, invariant genes with interquartile ranges (IQR) ≤0.15 for aphid, ≤0.15 for *B. aphidicola* and ≤ 0.75 for *H. defensa* were removed based on inspection of histograms. A gene correlation matrix with the Pearson’s correlation coefficient of each pairwise combination of IQR-filtered aphid and *H. defensa* genes was constructed. Genes were clustered by their expression patterns into modules using flashClust v.1.01–2 [107] and similar modules were combined after estimating an adequate number of clusters using the “gap” statistic implemented in cluster v.2.0.7–1 [108] and inspecting module correlation dendrograms. To test whether the pattern of gene clustering was due to random chance, the simprof similarity profile permutation test implemented in clustsig v 1.1 [109] was used. It created an expected data distribution from 100 similarity profiles and compared the observed test statistics to the null distribution based on 99 similarity profiles at α < 0.01. Module eigengenes were calculated with WGCNA v. 1.66 [105, 106] and were correlated in an eigengene correlation matrix as well as to titre (approximated by the *H. defensa* to aphid read ratio).

Both WGCNA and correlation gene modules were tested for enrichment of GO-terms using GoFuncR v1.6.1 [110] in R v3.6.3 and results were corrected for multiple testing and interdependency with 1000 replicates and an adjusted significance threshold of q < 0.01. *B. aphidicola* modules were additionally tested for enrichment of KEGG-pathways [111] with clusterprofiler v3.14.3 [112] in R v3.6.3. Both the WGNCA and correlation approach rely on several thresholds, which can be found in the adapted R scripts from Smith et al. [51] in Additional File 4.

### Index hopping

Even though aphids from treatment H0 were not infected by *H. defensa,* a small number of read pairs were assigned to *H. defensa* genes in H0 libraries. Notable examples are CdtB, which should be specific to isolate H402 but was also assembled in H15, H76 and H85, and YD-repeat toxin, which should be specific to H15, H76 and H85 but was also partially assembled in H402. This observation could be explained by contamination, misassignment of reads between genes that are conserved between *B. aphidicola* and *H. defensa*, or index hopping, with the latter being the most parsimonious explanation. Firstly, misassignment to conserved genes was not the cause: Highly expressed and strain-specific genes like the CdtB and YD-repeat toxin seemed to be expressed at low levels in *H. defensa* strains that did not contain these genes as well as in the ‘phantom’ *H. defensa* of H0 aphids*.* Secondly, index hopping was more likely than contamination for two reasons. A) Index hopping is expected to occur in sequencing assays like the one we used [113]. B) Contamination of every single library with foreign *H. defensa* is unlikely. We therefore accepted index hopping as the most parsimonious explanation.

We estimated that approximately 0.2% of read pairs had undergone index hopping, which falls just below the range of 0.3–0.5% expected by the manufacturer (Illumina Inc., 2018). Given evidence that all possible index hopping combinations occur at uniform distribution around the mean [114], the influence of index hopping on fold change values was considered negligible.

## Supplementary Information

**Additional file 1: Supplementary Fig. 1**. Library H15R1 is an outlier. Library H15R1 is located at the lower right corner of a PCA of the normalised and variance stabilisation transformed read count of all aphid genes. Aphid hosts were uninfected (H0, black) or infected (infecting strains H15 (blue), H402 (orange), H76 (grey) or H85 (red)) by *H. defensa*. **Supplementary Fig. 2**. Correlation of aphid and *H. defensa* gene modules. Pearson correlation coefficient between eigengenes of aphid and *H. defensa* modules. Coloured: Correlation has a *p*-value of < 0.01, red indicates positive and blue indicates negative correlation. **Supplementary Fig. 3**. Correlation of *B. aphidicola* and *H. defensa* gene modules. Pearson correlation coefficient between eigengenes of *B. aphidicola* and *H. defensa* modules. Coloured: Correlation has a p-value of < 0.01, red indicates positive and blue indicates negative correlation. **Supplementary Fig. 4**. High RNA integrity after total RNA extraction. Bioanalyzer 2100 electopherograms of all libraries in batch A and B. Comparison with Schroeder et al. (2006) implied a RIN of 8 for sample H0R2 and 9–10 for all others. Ribosomal RNA peaks of the different organisms are visible. Consider that a double 18S rRNA peak is expected for several insect species.

**Additional file 2: Supplementary Table 1**. Number of reads per organism. Number of reads in each library (‘reads processed’) and number and fraction of reads mapped to each organism by Kallisto. Column ‘tmt’ indicates treatment, i.e. uninfected (H0) or *H. defensa*-infected aphid hosts (infecting strains H15, H402, H76 or H85). ‘Batch’ indicates which libraries were grouped during RNA extraction and library preparation. The library that was excluded from analysis is marked with **. Read counts marked with * are most likely a result of index hopping. **Supplementary Table 2**. Read assignment to taxa. Fraction of reads assigned to different taxa by Kraken. Fractions were averaged over all libraries of each treatment, except for treatment H15 for which the strongly contaminated library H15R1 was listed separately from the other three libraries (H15*). Aphid hosts were uninfected (H0) or infected (infecting strains H15, H402, H76 or H85) by *H. defensa*. As this table contains only taxa of specific interest, the read fractions are not expected to add up to 100%. Fractions below 0.01 were not extracted from the Kraken output, which is indicated by “< 0.01”. **Supplementary Table 3**. Completeness of assembly. BUSCO analysis of the aphid transcripts and prokaryote genes. BUSCO scores were calculated in relation to the reference databases *Arthropoda*, *Insecta* and *Proteobacteria*. **Supplementary Table 4**. Overview over the aphid’s differential expression. Aphids infected with *H. defensa* (H15, H76, H85 or H402) were compared to aphids not infected by *H. defensa *(H0). In a reduced model containing only libraries of treatment H15 and H85, differential gene expression of aphids in presence of these two closely related *H. defensa* strains was analysed. For each pairwise comparison, number of differentially expressed genes (DEG), median fold changes of upregulated and downregulated genes as well as maximum fold change of upregulated and downregulated genes are indicated. No GO-terms were found enriched among the DEG. **Supplementary Table 5**. Differential expression of aphid genes (full model). Results of differential expression analysis comparing gene expression of aphids infected with *H. defensa* (H15, H76, H85 or H402) to aphids not infected by *H. defensa* (H0). *P*-values adjusted for multiple testing (padj) < 0.01 and absolute log2 fold changes(L2FC) > 0.5 are indicated by coloured backgrounds. **Supplementary Table 6**. Differential expression of aphid genes (reduced model). Results of differential expression analysis comparing gene expression of aphids infected with *H. defensa* H15 to aphids infected with *H. defensa* H85. P-values adjusted for multiple testing (padj) < 0.01 and absolute log2 fold changes (L2FC) > 0.5 are indicated by coloured backgrounds. **Supplementary Table 7**. Differential expression of *H. defensa* genes (full model). Results of differential expression analysis comparing gene expression of *H. defensa* strains H15, H76 or H402 to *H. defensa* strain H85 (full model with all strains). P-values adjusted for multiple testing (padj) < 0.01 and absolute log2 fold changes (L2FC) > 0.5 are indicated by coloured backgrounds. **Supplementary Table 8**. Differential expression of *B. aphidicola* genes. Results of differential expression analysis comparing gene expression of *B. aphidicola* in aphid hosts infected with *H. defensa* (H15, H76, H85 or H402) to uninfected aphid hosts (H0). P-values adjusted for multiple testing (padj) < 0.01 and absolute log2 fold changes (L2FC) > 0.5 are indicated by coloured backgrounds. For each gene, read counts in each replicate are indicated. **Supplementary Table 9**. Correlation of aphid gene expression. Correlation coefficient and *p*-values of aphid gene modules with *H. defensa* titre and toxin type. Coloured: Correlation has a p-value of < 0.01. If no GO-terms are enriched in a module, the value in ‘associated GO-terms’ is set to ‘NA’. a) WGCNA analysis with aphid and *H. defensa* genes. b) Correlation analysis with aphid and *H. defensa* genes. **Supplementary Table 10**. Correlation of *H. defensa* gene expression. Correlation coefficient and p-values of *H. defensa* gene modules with *H. defensa* titre and toxin type. Coloured: Correlation has a p-value of < 0.01. If no GO-terms are enriched in a module, the value in ‘associated GO-terms’ is set to ‘NA’. a) WGCNA analysis with aphid and *H. defensa* genes. b) Correlation analysis with aphid and *H. defensa* genes. c) Correlation analysis with *B. aphidicola *and *H. defensa* genes. **Supplementary Table 11**. Correlation of *B. aphidicola* gene expression. Correlation coefficient and p-values of *B. aphidicola* gene modules with *H. defensa* titre and toxin type. Coloured: Correlation has a p-value of < 0.01. If no GO-terms or KEGG pathways are enriched in a module, the values in ‘associated GO-terms’ and ‘associated KEGG pathways’ is set to ‘NA’. a) WGCNA analysis with *B. aphidicola* and *H. defensa* genes. b) Correlation analysis with *B. aphidicola* and *H. defensa* genes. **Supplementary Table 12**. Origin of aphid clones. Collection date, site and host plant for the aphid clones used as donors and recipients during the transfections that created the infected A06–407 sublines. **Supplementary Table 13**. Accession numbers of *H. defensa* assemblies. Protein fasta files from genome assemblies of *H. defensa* were downloaded from NCBI and provided to Prokka for gene prediction and to BUSCO for phylogenetic analyses. **Supplementary Table 14**. Accession numbers of contaminant bacterial genomes. Coding DNA sequences (CDS) from genome assemblies of the most frequent contaminant bacteria were downloaded from NCBI and provided to Kallisto during mapping.

**Additional File 3 **Module membership and associated GO-terms for aphid, *H. defensa* and *B. aphidicola* genes.

**Additional File 4.** R-scripts of the correlation approach and WGCNA analysis adapted from Smith et al. [51].

## Data Availability

The datasets supporting the conclusions of this article are available in the GenBank repository, at http://www.ncbi.nlm.nih.gov/biosample under BioSample ID no. SAMN10606880 to SAMN10606919, and in the Dryad repository, at 10.5061/dryad.cc2fqz667.

## References

[1] Sanchez-Contreras M, Vlisidou I (2008). The diversity of insect-bacteria interactions and its applications for disease control. Biotechnol Genet Eng Rev.

[2] Ratzka C, Gross R, Feldhaar H (2012). Endosymbiont tolerance and control within insect hosts. Insects.

[3] Brinza L, Viñuelas J, Cottret L, Calevro F, Rahbé Y, Febvay G, et al. Systemic analysis of the symbiotic function of *Buchnera aphidicola*, the primary endosymbiont of the pea aphid *Acyrthosiphon pisum*. Comptes Rendus Biol. 2009;332(11):1034–49. 10.1016/j.crvi.2009.09.007. 10.1016/j.crvi.2009.09.007 19909925

[4] Moran N, Baumann P (1994). Phylogenetics of cytoplasmically inherited microorganisms of arthropods. Trends Ecol Evol.

[5] Hansen AK, Moran NA (2011). Aphid genome expression reveals host-symbiont cooperation in the production of amino acids. Proc Natl Acad Sci U S A.

[6] Douglas AE. Nutritional interactions in insect-microbial symbioses: aphids and their symbiotic bacteria *Buchnera*. Annu Rev Entomol. 1998;43(1):17–37. 10.1146/annurev.ento.43.1.17. 10.1146/annurev.ento.43.1.17 15012383

[7] Wilkinson TL, Fukatsu T, Ishikawa H. Transmission of symbiotic bacteria *Buchnera *to parthenogenetic embryos in the aphid *Acyrthosiphon pisum* (Hemiptera: Aphidoidea). Arthropod Struct Dev. 2003;32(2–3):241–5. 10.1016/S1467-8039(03)00036-7. 10.1016/S1467-8039(03)00036-7 18089009

[8] Oliver KM, Degnan PH, Burke GR, Moran NA (2010). Facultative symbionts in aphids and the horizontal transfer of ecologically important traits. Annu Rev Entomol.

[9] Moran NA, Russell JA, Koga R, Fukatsu T. Evolutionary relationships of three new species of *Enterobacteriaceae *living as symbionts of aphids and other insects. Appl Environ Microbiol. 2005;71(6):3302–10. 10.1128/AEM.71.6.3302-3310.2005. 10.1128/AEM.71.6.3302-3310.2005 PMC1151865 15933033

[10] Gehrer L, Vorburger C (2012). Parasitoids as vectors of facultative bacterial endosymbionts in aphids. Biol Lett.

[11] Pons I, Renoz F, Noël C, Hance T. Circulation of the cultivable symbiont *Serratia symbiotica* in aphids is mediated by plants. Front Microbiol. 2019;10(764):1–13. 10.3389/fmicb.2019.00764 PMC6476230 31037067

[12] Lemaitre B, Hoffmann J. The host defense of *Drosophila melanogaster*. Annu Rev Immunol. 2007;25(1):697–743. 10.1146/annurev.immunol.25.022106.141615. 10.1146/annurev.immunol.25.022106.141615 17201680

[13] Gerardo NM, Altincicek B, Anselme C, Atamian H, Barribeau SM, de Vos M, Duncan EJ, Evans JD, Gabaldon T, Ghanim M (2010). Immunity and other defenses in pea aphids*, Acyrthosiphon pisum*. Genome Biol.

[14] Laughton AM, Garcia JR, Altincicek B, Strand MR, Gerardo NM (2011). Characterisation of immune responses in the pea aphid*, Acyrthosiphon pisum*. J Insect Physiol.

[15] Guo J, Hatt S, He K, Chen J, Francis F, Wang Z (2017). Nine facultative endosymbionts in aphids. A review. J Asia Pacific Entomol.

[16] Barribeau SM, Parker BJ, Gerardo NM (2014). Exposure to natural pathogens reveals costly aphid response to fungi but not bacteria. Ecol Evol.

[17] Altincicek B, ter Braak B, Laughton AM, Udekwu KI, Gerardo NM. *Escherichia coli* K-12 pathogenicity in the pea aphid, *Acyrthosiphon pisum*, reveals reduced antibacterial defense in aphids. Dev Comp Immunol. 2011;35(10):1091–7. 10.1016/j.dci.2011.03.017. 10.1016/j.dci.2011.03.017 21527277

[18] Renoz F, Noel C, Errachid A, Foray V, Hance T. Infection dynamic of symbiotic bacteria in the pea aphid *Acyrthosiphon pisum* gut and host immune response at the early steps in the infection process. PLoS One. 2015;10(3):e0122099. 10.1371/journal.pone.0122099. 10.1371/journal.pone.0122099 PMC4374939 25811863

[19] Schmitz A, Anselme C, Ravallec M, Rebuf C, Simon J-C, Gatti J-L, Poirié M (2012). The cellular immune response of the pea aphid to foreign intrusion and symbiotic challenge. PLoS One.

[20] Scarborough CL, Ferrari J, Godfray HCJ (2005). Aphid protected from pathogen by endosymbiont. Science.

[21] Oliver KM, Moran NA, Hunter MS (2005). Variation in resistance to parasitism in aphids is due to symbionts not host genotype. Proc Natl Acad Sci U S A.

[22] Wagner SM, Martinez AJ, Ruan Y-M, Kim KL, Lenhart PA, Dehnel AC, Oliver KM, White JA (2015). Facultative endosymbionts mediate dietary breadth in a polyphagous herbivore. Funct Ecol.

[23] Russell JA, Moran NA (2006). Costs and benefits of symbiont infection in aphids: variation among symbionts and across temperatures. Proc R Soc B.

[24] Smith AH, Łukasik P, O'Connor MP, Lee A, Mayo G, Drott MT, Doll S, Tuttle R, Disciullo RA, Messina A, Oliver KM, Russell JA (2015). Patterns, causes and consequences of defensive microbiome dynamics across multiple scales. Mol Ecol.

[25] Vorburger C, Rouchet R (2016). Are aphid parasitoids locally adapted to the prevalence of defensive symbionts in their hosts?. BMC Evol Biol.

[26] Dykstra HR, Weldon SR, Martinez AJ, White JA, Hopper KR, Heimpel GE, et al. Factors limiting the spread of the protective symbiont *Hamiltonella defensa* in *Aphis craccivora* aphids. Appl Environ Microbiol. 2014;80(18):5818–27. 10.1128/AEM.01775-14. 10.1128/AEM.01775-14 PMC4178609 25015890

[27] Oliver KM, Campos J, Moran NA, Hunter MS (2008). Population dynamics of defensive symbionts in aphids. Proc R Soc B.

[28] Hafer-Hahmann N, Vorburger C (2020). Parasitoids as drivers of symbiont diversity in an insect host. Ecol Lett.

[29] Price, D.R.G., H. Feng, J.D. Baker, S. Bavan, C.W. Luetje and A.C.C. Wilson, Aphid amino acid transporter regulates glutamine supply to intracellular bacterial symbionts*.* Proc Natl Acad Sci U S A, 2014. 111(1): p. 320–325, 1, DOI: 10.1073/pnas.1306068111. 10.1073/pnas.1306068111 PMC3890774 24367072

[30] Colella S, Parisot N, Simonet P, Gaget K, Duport G, Baa-Puyoulet P, et al. Bacteriocyte reprogramming to cope with nutritional stress in a phloem sap feeding Hemipteran, the pea aphid *Acyrthosiphon pisum*. Front Physiol. 2018;9(1498). 10.3389/fphys.2018.01498 PMC6209921 30410449

[31] Stoy KS, Gibson AK, Gerardo NM, Morran LT (2020). A need to consider the evolutionary genetics of host–symbiont mutualisms. J Evol Biol.

[32] Oliver KM, Russell JA, Moran NA, Hunter MS (2003). Facultative bacterial symbionts in aphids confer resistance to parasitic wasps. Proc Natl Acad Sci.

[33] Vorburger C, Sandrock C, Gouskov A, Castañeda LE, Ferrari J (2009). Genotypic variation and the role of defensive endosymbionts in an all-parthenogenetic host-parasitoid interaction. Evolution.

[34] Schmid M, Sieber R, Zimmermann Y-S, Vorburger C (2012). Development, specificity and sublethal effects of symbiont-conferred resistance to parasitoids in aphids. Funct Ecol.

[35] Moran NA, Degnan PH, Santos SR, Dunbar HE, Ochman H (2005). The players in a mutualistic symbiosis: insects, bacteria, viruses, and virulence genes. Proc Natl Acad Sci U S A.

[36] Oliver KM, Degnan PH, Hunter MS, Moran NA (2009). Bacteriophages encode factors required for protection in a symbiotic mutualism. Science.

[37] Degnan PH, Moran NA (2008). Diverse phage-encoded toxins in a protective insect endosymbiont. Appl Environ Microbiol.

[38] Oliver KM, Higashi CH. Variations on a protective theme: *Hamiltonella defensa* infections in aphids variably impact parasitoid success. Curr Opin Insect Sci. 2019;32:1–7. 10.1016/j.cois.2018.08.009. 10.1016/j.cois.2018.08.009 31113620

[39] Weldon SR, Strand MR, Oliver KM. Phage loss and the breakdown of a defensive symbiosis in aphids. Proc R Soc B Biol Sci. 2013;280(1751). 10.1098/rspb.2012.2103 PMC3574403 23193123

[40] Vorburger C, Gouskov A (2011). Only helpful when required: a longevity cost of harbouring defensive symbionts. J Evol Biol.

[41] Vorburger C, Perlman SJ (2018). The role of defensive symbionts in host–parasite coevolution. Biol Rev.

[42] Cayetano L, Rothacher L, Simon J-C, Vorburger C. Cheaper is not always worse: Strongly protective isolates of a defensive symbiont are less costly to the aphid host. Proc R Soc B. 2015;282(1799). 10.1098/rspb.2014.2333 PMC4286048 25473015

[43] Dennis AB, Patel V, Oliver KM, Vorburger C (2017). Parasitoid gene expression changes after adaptation to symbiont-protected hosts. Evolution.

[44] Kaech H, Vorburger C. Horizontal transmission of the heritable protective endosymbiont *Hamiltonella defensa* depends on titre and haplotype. Front Microbiol. 2021;11:628755. 10.3389/fmicb.2020.628755. 10.3389/fmicb.2020.628755 PMC7840887 33519791

[45] Wenger JA, Cassone BJ, Legeai F, Johnston JS, Bansal R, Yates AD, et al. Whole genome sequence of the soybean aphid, *Aphis glycines*. Insect Biochem Mol Biol. 2017(102917):1–10. 10.1016/j.ibmb.2017.01.005 28119199

[46] Goto A, Kumagai T, Kumagai C, Hirose J, Narita H, Mori H, et al. A *Drosophila* haemocyte-specific protein, hemolectin, similar to human von Willebrand factor. Biochem J. 2001;359(Pt 1):99–108. 10.1042/bj3590099. 10.1042/0264-6021:3590099 PMC1222125 11563973

[47] Lebestky T, Chang T, Hartenstein V, Banerjee U. Specification of *Drosophila* hematopoietic lineage by conserved transcription factors. Science. 2000;288(5463):146–9. 10.1126/science.288.5463.146. 10.1126/science.288.5463.146 10753120

[48] Franc NC, Dimarcq J-L, Lagueux M, Hoffmann J, Ezekowitz RAB. Croquemort, a novel *Drosophila* hemocyte/macrophage receptor that recognizes apoptotic cells. Immunity. 1996;4(5):431–43. 10.1016/S1074-7613(00)80410-0. 10.1016/s1074-7613(00)80410-0 8630729

[49] Zanet J, Stramer B, Millard T, Martin P, Payre F, Plaza S. Fascin is required for blood cell migration during *Drosophila* embryogenesis. Development. 2009;136(15):2557–65. 10.1242/dev.036517. 10.1242/dev.036517 19592575

[50] Nelson RE, Fessler LI, Takagi Y, Blumberg B, Keene DR, Olson PF, et al. Peroxidasin: a novel enzyme-matrix protein of *Drosophila* development. EMBO J. 1994;13(15):3438–47. 10.1002/j.1460-2075.1994.tb06649.x. 10.1002/j.1460-2075.1994.tb06649.x PMC395246 8062820

[51] Smith TE, Moran NA. Coordination of host and symbiont gene expression reveals a metabolic tug-of-war between aphids and *Buchnera*. Proc Natl Acad Sci. 2020;117(4):2113–21. 10.1073/pnas.1916748117. 10.1073/pnas.1916748117 PMC6995025 31964845

[52] Chevignon G, Boyd BM, Brandt JW, Oliver KM, Strand MR. Culture-facilitated comparative genomics of the facultative Symbiont *Hamiltonella defensa*. Genome Biol Evol. 2018;10(3):786–802. 10.1093/gbe/evy036. 10.1093/gbe/evy036 PMC5841374 29452355

[53] Senti K-A, Brennecke J. The piRNA pathway: a fly's perspective on the guardian of the genome. Trends Genet. 2010;26(12):499–509. 10.1016/j.tig.2010.08.007. 10.1016/j.tig.2010.08.007 PMC4988489 20934772

[54] Rubio-Texeira M. Urmylation controls *Nil1p *and *Gln3p*-dependent expression of nitrogen-catabolite repressed genes in *Saccharomyces cerevisiae*. FEBS Lett. 2007;581(3):541–50. 10.1016/j.febslet.2007.01.019. 10.1016/j.febslet.2007.01.019 17254574

[55] Khoshnood B, Dacklin I, Grabbe C. *Urm1*: an essential regulator of JNK signaling and oxidative stress in *Drosophila melanogaster*. Cell Mol Life Sci. 2016;73(9):1939–54. 10.1007/s00018-015-2121-x. 10.1007/s00018-015-2121-x PMC11108535 26715182

[56] Ma L, Liu L, Zhao Y, Yang L, Chen C, Li Z, Lu Z (2020). JNK pathway plays a key role in the immune system of the pea aphid and is regulated by microRNA-184. PLoS Pathog.

[57] Kotani E, Yamakawa M, Iwamoto S, Tashiro M, Mori H, Sumida M, Matsubara F, Taniai K, Kadono-Okuda K, Kato Y (1995). Cloning and expression of the gene hemocytin, an insect humoral lectin which is homologous with the mammalian von Willebrand factor. Biochim Biophys Acta.

[58] Goto A, Kadowaki T, Kitagawa Y. *Drosophila *hemolectin gene is expressed in embryonic and larval hemocytes and its knock down causes bleeding defects. Dev Biol. 2003;264(2):582–91. 10.1016/j.ydbio.2003.06.001. 10.1016/j.ydbio.2003.06.001 14651939

[59] Scherfer C, Karlsson C, Loseva O, Bidla G, Goto A, Havemann J, et al. Isolation and characterization of hemolymph clotting factors in *Drosophila melanogaster* by a pullout method. Curr Biol. 2004;14(7):625–9. 10.1016/j.cub.2004.03.030. 10.1016/j.cub.2004.03.030 15062105

[60] Eleftherianos I, Revenis C (2011). Role and importance of phenoloxidase in insect hemostasis. J Innate Immun.

[61] Luo C, Belghazi M, Schmitz A, Lemauf S, Desneux N, Simon J-C, et al. Hosting certain facultative symbionts modulates the phenoloxidase activity and immune response of the pea aphid *Acyrthosiphon pisum*. Insect Sci. 2020; n/a(n/a). 10.1111/1744-7917.12888 33200579

[62] Sanchez Bosch P, Makhijani K, Herboso L, Gold KS, Baginsky R, Woodcock KJ, et al. Adult *Drosophila *lack hematopoiesis but rely on a blood cell reservoir at the respiratory epithelia to relay infection signals to surrounding tissues. Dev Cell. 2019;51(6):787–803 e5. 10.1016/j.devcel.2019.10.017 PMC7263735 31735669

[63] Nichols HL, Goldstein EB, Ziabari OS, Parker BJ. Intraspecific variation in immune gene expression and heritable symbiont density. bioRxiv. 2020; 2020.12.17.420083. 10.1371/journal.ppat.1009552 PMC8102006 33901257

[64] Kutsukake M, Moriyama M, Shigenobu S, Meng X-Y, Nikoh N, Noda C, Kobayashi S, Fukatsu T (2019). Exaggeration and cooption of innate immunity for social defense. Proc Natl Acad Sci.

[65] Degnan PH, Yu Y, Sisneros N, Wing RA, Moran NA. *Hamiltonella defensa*, genome evolution of protective bacterial endosymbiont from pathogenic ancestors. Proc Natl Acad Sci U S A. 2009;106(22):9063–8. 10.1073/pnas.0900194106. 10.1073/pnas.0900194106 PMC2690004 19451630

[66] Wilcox JL, Dunbar HE, Wolfinger RD, Moran NA (2003). Consequences of reductive evolution for gene expression in an obligate endosymbiont. Mol Microbiol.

[67] Moran NA, Dunbar HE, Wilcox JL. Regulation of transcription in a reduced bacterial genome: nutrient-provisioning genes of the obligate symbiont *Buchnera aphidicola*. J Bacteriol. 2005;187(12):4229–37. 10.1128/JB.187.12.4229-4237.2005. 10.1128/JB.187.12.4229-4237.2005 PMC1151715 15937185

[68] Rouïl J, Jousselin E, Coeur d’Acier A, Cruaud C, Manzano-Marín A. The protector within: comparative genomics of APSE phages across aphids reveals rampant recombination and diverse toxin arsenals. Genome Biol Evol. 2020;12(6):878–89. 10.1093/gbe/evaa089 PMC7313666 32386316

[69] van der Wilk F, Dullemans AM, Verbeek M, van den Heuvel JFJM. Isolation and characterization of APSE-1, a bacteriophage infecting the secondary endosymbiont of *Acyrthosiphon pisum*. Virology. 1999;262(1):104–13. 10.1006/viro.1999.9902. 10.1006/viro.1999.9902 10489345

[70] Gasch AP, Spellman PT, Kao CM, Carmel-Harel O, Eisen MB, Storz G, Botstein D, Brown PO (2000). Genomic expression programs in the response of yeast cells to environmental changes. Mol Biol Cell.

[71] Tao H, Bausch C, Richmond C, Blattner FR, Conway T. Functional genomics: expression analysis *Escherichia coli* growing on minimal and rich media. J Bacteriol. 1999;181(20):6425–40. 10.1128/JB.181.20.6425-6440.1999. 10.1128/jb.181.20.6425-6440.1999 PMC103779 10515934

[72] Zhou A, He Z, Redding-Johanson AM, Mukhopadhyay A, Hemme CL, Joachimiak MP, et al. Hydrogen peroxide-induced oxidative stress responses in *Desulfovibrio vulgaris* Hildenborough. Environ Microbiol. 2010;12(10):2645–57. 10.1111/j.1462-2920.2010.02234.x. 10.1111/j.1462-2920.2010.02234.x 20482586

[73] Aseev LV, Koledinskaya LS, Boni IV. Regulation of ribosomal protein operons *rplM-rpsI*, *rpmB-rpmG*, and *rplU-rpmA* at the transcriptional and translational levels. J Bacteriol. 2016;198(18):2494–502. 10.1128/JB.00187-16. 10.1128/JB.00187-16 PMC4999927 27381917

[74] Zhou X, Liao W-J, Liao J-M, Liao P, Lu H (2015). Ribosomal proteins: functions beyond the ribosome. J Mol Cell Biol.

[75] Bolger AM, Lohse M, Usadel B. Trimmomatic: a flexible trimmer for Illumina sequence data. Bioinformatics. 2014;30(15):2114–20. 10.1093/bioinformatics/btu170. 10.1093/bioinformatics/btu170 PMC4103590 24695404

[76] Andrews S (2010). FastQC: a quality control tool for high throughput sequence data.

[77] Wood DE, Salzberg SL (2014). Kraken: ultrafast metagenomic sequence classification using exact alignments. Genome Biol.

[78] Grabherr MG, Haas BJ, Yassour M, Levin JZ, Thompson DA, Amit I, Adiconis X, Fan L, Raychowdhury R, Zeng Q, Chen Z, Mauceli E, Hacohen N, Gnirke A, Rhind N, di Palma F, Birren BW, Nusbaum C, Lindblad-Toh K, Friedman N, Regev A (2011). Trinity: reconstructing a full-length transcriptome without a genome from RNA-Seq data. Nat Biotechnol.

[79] Fu L, Niu B, Zhu Z, Wu S, Li W (2012). CD-HIT: accelerated for clustering the next-generation sequencing data. Bioinformatics.

[80] Schmieder R, Lim YW, Edwards R (2012). Identification and removal of ribosomal RNA sequences from metatranscriptomes. Bioinformatics.

[81] Schmieder R, Edwards R (2011). Quality control and preprocessing of metagenomic datasets. Bioinformatics.

[82] Camacho C, Coulouris G, Avagyan V, Ma N, Papadopoulos J, Bealer K, Madden TL (2009). BLAST+: architecture and applications. BMC Bioinformatics.

[83] Altschul SF, Madden TL, Schäffer AA, Zhang J, Zhang Z, Miller W, Lipman DJ (1997). Gapped BLAST and PSI-BLAST: a new generation of protein database search programs. Nucleic Acids Res.

[84] Buchfink B, Xie C, Huson DH (2014). Fast and sensitive protein alignment using DIAMOND. Nat Methods.

[85] BioBam Bioinformatics, OmicsBox – Bioinformatics Made Easy. https://www.biobam.com/omicsbox. Accessed 3 Mar 2019.

[86] Conway T, Creecy JP, Maddox SM, Grissom JE, Conkle TL, Shadid TM, Teramoto J, San Miguel P, Shimada T, Ishihama A (2014). Unprecedented high-resolution view of bacterial operon architecture revealed by RNA Sequencing. mBio.

[87] Kearse M, Moir R, Wilson A, Stones-Havas S, Cheung M, Sturrock S, Buxton S, Cooper A, Markowitz S, Duran C, Thierer T, Ashton B, Meintjes P, Drummond A (2012). Geneious basic: an integrated and extendable desktop software platform for the organization and analysis of sequence data. Bioinformatics.

[88] Seemann T (2014). Prokka: rapid prokaryotic genome annotation. Bioinformatics.

[89] Price A, Gibas C (2017). The quantitative impact of read mapping to non-native reference genomes in comparative RNA-Seq studies. PLoS One.

[90] Bray NL, Pimentel H, Melsted P, Pachter L (2016). Near-optimal probabilistic RNA-seq quantification. Nat Biotechnol.

[91] Love MI, Huber W, Anders S (2014). Moderated estimation of fold change and dispersion for RNA-seq data with DESeq2. Genome Biol.

[92] Soneson C, Love M, Robinson M. Differential analyses for RNA-seq: Transcript-level estimates improve gene-level inferences [version 2; peer review: 2 approved]. F1000Research. 2016;4(1521). 10.12688/f1000research.7563.1 PMC4712774 26925227

[93] R Core Team (2018). R: A language and environment for statistical computing.

[94] Dillies M-A, Rau A, Aubert J, Hennequet-Antier C, Jeanmougin M, Servant N, Keime C, Marot G, Castel D, Estelle J, Guernec G, Jagla B, Jouneau L, Laloe D, le Gall C, Schaeffer B, le Crom S, Guedj M, Jaffrezic F, on behalf of The French StatOmique Consortium (2013). A comprehensive evaluation of normalization methods for Illumina high-throughput RNA sequencing data analysis. Brief Bioinform.

[95] Lex A, Gehlenborg N, Strobelt H, Vuillemot R, Pfister H (2014). UpSet: visualization of intersecting sets. IEEE Trans Vis Comput Graph.

[96] Marini F, Binder H (2019). pcaExplorer: an R/Bioconductor package for interacting with RNA-seq principal components. BMC Bioinformatics.

[97] Götz S, García-Gómez JM, Terol J, Williams TD, Nagaraj SH, Nueda MJ, Robles M, Talón M, Dopazo J, Conesa A (2008). High-throughput functional annotation and data mining with the Blast2GO suite. Nucleic Acids Res.

[98] Kriventseva EV, Simão FA, Klioutchnikov G, Seppey M, Manni M, Ioannidis P, Waterhouse RM, Zdobnov EM (2017). BUSCO applications from quality assessments to gene prediction and Phylogenomics. Mol Biol Evol.

[99] Kriventseva EV, Zdobnov EM, Simão FA, Ioannidis P, Waterhouse RM (2015). BUSCO: assessing genome assembly and annotation completeness with single-copy orthologs. Bioinformatics.

[100] Katoh K, Standley DM (2013). MAFFT multiple sequence alignment software version 7: improvements in performance and usability. Mol Biol Evol.

[101] Silla-Martínez JM, Capella-Gutiérrez S, Gabaldón T (2009). trimAl: a tool for automated alignment trimming in large-scale phylogenetic analyses. Bioinformatics.

[102] Lanfear R, Frandsen PB, Wright AM, Senfeld T, Calcott B. PartitionFinder 2: New methods for selecting partitioned models of evolution for molecular and morphological phylogenetic analyses. Mol Biol Evol. 2016;34(3). 10.1093/molbev/msw260 28013191

[103] Stamatakis A (2014). RAxML version 8: a tool for phylogenetic analysis and post-analysis of large phylogenies. Bioinformatics.

[104] Lanfear R, Calcott B, Kainer D, Mayer C, Stamatakis A. Selecting optimal partitioning schemes for phylogenomic datasets. BMC Evol Biol. 2014;14(1). 10.1186/1471-2148-14-82 PMC4012149 24742000

[105] Zhang B, Horvath S. A general framework for weighted co-expression network analysis. Appl. Genet. Mol. Biol. 2005;4(Article 17). 10.2202/1544-6115.1128 16646834

[106] Langfelder P, Horvath S (2008). WGCNA: an R package for weighted correlation network analysis. BMC Bioinformatics.

[107] Langfelder P, Horvath S (2012). Fast R functions for robust correlations and hierarchical clustering. J Stat Softw.

[108] Maechler M, Rousseeuw P, Struyf A, Hubert M, Hornik K (2018). cluster: cluster analysis basics and extensions. R package version 2.0.7–1.

[109] Whitaker D, Christman M (2014). Clustsig: Significant cluster analysis. R package version 1.1.

[110] Grote S (2020). GOfuncR: Gene ontology enrichment using FUNC. R package version 1.8.0.

[111] Kanehisa M, Goto S (2000). KEGG: Kyoto encyclopedia of genes and genomes. Nucleic Acids Res.

[112] Yu G, Wang L-G, Han Y, He Q-Y (2012). clusterProfiler: an R package for comparing biological themes among gene clusters. OMICS.

[113] Illumina Inc. Effects of index misassignment on multiplexing and downstream analysis. 2018 [cited 2020 February]; 770–2017-004-D:[Available from: https://emea.illumina.com/content/dam/illumina-marketing/documents/products/whitepapers/index-hopping-white-paper-770-2017-004.pdf.

[114] Costello M, Fleharty M, Abreu J, Farjoun Y, Ferriera S, Holmes L, Granger B, Green L, Howd T, Mason T, Vicente G, Dasilva M, Brodeur W, DeSmet T, Dodge S, Lennon NJ, Gabriel S (2018). Characterization and remediation of sample index swaps by non-redundant dual indexing on massively parallel sequencing platforms. BMC Genomics.

