# Supplementary material for: Triple RNA-Seq characterizes aphid gene expression in response to infection with unequally virulent strains of the endosymbiont Hamiltonella defensa
Source: BMC Genomics. 2021 Jun 16;22:449. doi: 10.1186/s12864-021-07742-8 (PMC8207614; doi:10.1186/s12864-021-07742-8)
Supplement: Supplementary file 1 — Additional file 1: Supplementary Fig. 1. Library H15R1 is an outlier. Library H15R1 is located at the lower right corner of a PCA of the normalised and variance stabilisation transformed read count of all aphid genes. Aphid hosts were uninfected (H0, black) or infected (infecting strains H15 (blue), H402 (orange), H76 (grey) or H85 (red)) by H. defensa. Supplementary Fig. 2. Correlation of aphid and H. defensa gene modules. Pearson correlation coefficient between eigengenes of aphid and H. defensa modules. Coloured: Correlation has a p-value of < 0.01, red indicates positive and blue indicates negative correlation. Supplementary Fig. 3. Correlation of B. aphidicola and H. defensa gene modules. Pearson correlation coefficient between eigengenes of B. aphidicola and H. defensa modules. Coloured: Correlation has a p-value of < 0.01, red indicates positive and blue indicates negative correlation. Supplementary Fig. 4. High RNA integrity after total RNA extraction. Bioanalyzer 2100 electopherograms of all libraries in batch A and B. Comparison with Schroeder et al. (2006) implied a RIN of 8 for sample H0R2 and 9–10 for all others. Ribosomal RNA peaks of the different organisms are visible. Consider that a double 18S rRNA peak is expected for several insect species. [file 12864_2021_7742_MOESM1_ESM.pdf]

Triple RNA-Seq characterizes aphid gene expression in response to infection with unequally virulent strains of the endosymbiont *Hamiltonella defensa*

Heidi Kaech[1,2], Alice B. Dennis[3], Christoph Vorburger[1,2]

[1]Eawag, Swiss Federal Institute of Aquatic Science and Technology, Dübendorf, Switzerland

[2]D-USYS, Department of Environmental Systems Science, ETH Zürich, Switzerland

[3]Institute of Biochemistry and Biology, University Potsdam, Germany

Article Type: Research Article

Corresponding Author:

Heidi Kaech, Eawag – Eco, Überlandstrasse 133, 8600 Dübendorf, Switzerland

kaechh@outlook.com, phone +41 79 835 80 18

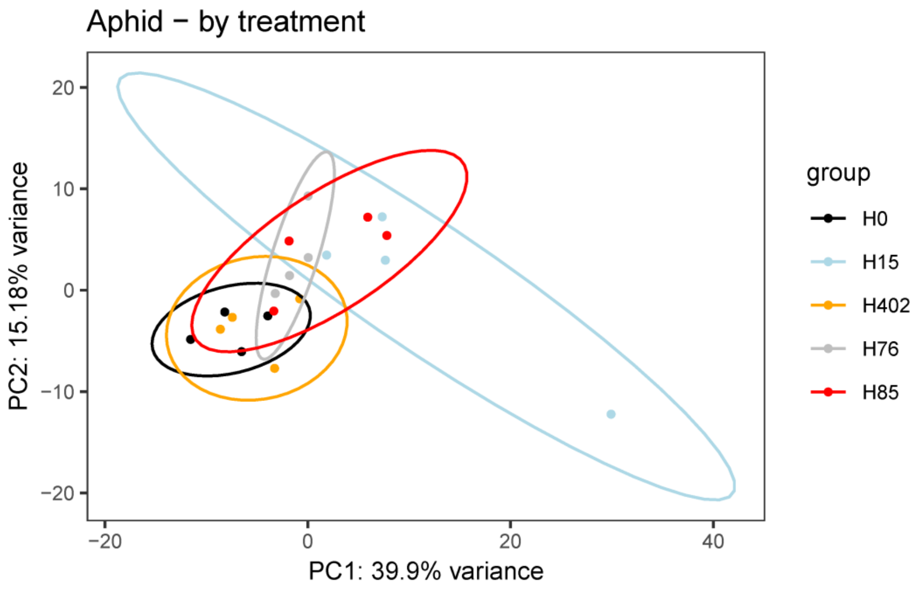

Additional File1, Supplementary Figure 1 – Library H15R1 is an outlier. Library H15R1 is located at the lower right corner of a PCA of the normalised and variance stabilisation transformed read count of all aphid genes. Aphid hosts were uninfected (H0, black) or infected (infecting strains H15 (blue), H402 (orange), H76 (grey) or H85 (red)) by *H. defensa*.

			Hamiltonella defensa												
			ApHdef-H10	ApHdef-H1	ApHdef-H11	ApHdef-H8	ApHdef-H7	ApHdef-H12	ApHdef-H9	ApHdef-H4	ApHdef-H2	ApHdef-H5	ApHdef-H3	ApHdef-H6	ApHdef-H13
		# genes	136	60	240	82	48	21	46	44	176	46	20	171	152
Aphis fabae	ApHdef-A2	141	0.08	0.36	-0.60	-0.58	0.77	-0.82	0.58	-0.39	0.33	-0.26	0.03	0.64	-0.81
	ApHdef-A4	243	-0.46	0.39	-0.91	-0.16	0.81	-0.49	0.08	-0.62	0.74	0.24	0.01	0.92	-0.73
	ApHdef-A5	99	-0.36	-0.02	-0.59	0.10	0.57	-0.24	-0.00	-0.68	0.45	0.34	-0.33	0.61	-0.32
	ApHdef-A7	383	0.46	-0.27	0.85	0.06	-0.76	0.43	-0.02	0.65	-0.70	-0.31	0.09	-0.86	0.62
	ApHdef-A6	22	-0.01	-0.69	0.01	0.36	0.33	-0.16	0.04	-0.69	-0.13	0.32	-0.85	-0.00	0.31
	ApHdef-A10	42	-0.46	0.30	-0.38	0.14	-0.06	0.42	-0.27	-0.05	0.45	0.25	0.21	0.37	-0.18
	ApHdef-A3	22	-0.90	0.42	-0.68	0.44	0.15	0.40	-0.71	-0.23	0.85	0.66	0.30	0.65	-0.20
	ApHdef-A1	42	0.73	0.41	0.23	-0.89	-0.13	-0.39	0.82	0.52	-0.40	-0.90	0.44	-0.18	-0.48
	ApHdef-A8	97	-0.09	-0.23	0.23	0.31	-0.39	0.56	-0.23	0.06	-0.12	0.16	-0.14	-0.24	0.39
	ApHdef-A9	121	-0.05	-0.06	-0.12	-0.01	0.08	0.06	0.09	-0.18	0.07	0.03	-0.16	0.13	-0.07
	ApHdef-A11	109	0.46	0.08	0.58	-0.27	-0.55	0.20	0.19	0.70	-0.49	-0.50	0.35	-0.58	0.20

Additional File1, Supplementary Figure 2 – Correlation of aphid and *H. defensa* gene modules. Pearson correlation coefficient between eigengenes of aphid and *H. defensa* modules. Coloured: Correlation has a p-value of <0.01, red indicates positive and blue indicates negative correlation.

			Hamiltonella defensa										
			BapHdef-H6	BapHdef-H8	BapHdef-H7	BapHdef-H2	BapHdef-H1	BapHdef-H3	BapHdef-H4	BapHdef-H10	BapHdef-H5	BapHdef-H11	BapHdef-H9
		# genes	60	136	240	48	21	46	44	176	171	82	152
Buchnera aphidicola	BapHdef-B2	11	0.11	-0.15	-0.24	-0.61	-0.15	-0.27	-0.20	0.25	0.35	0.31	-0.32
	BapHdef-B3	22	0.41	-0.36	0.18	-0.54	-0.18	0.21	-0.56	-0.28	0.53	-0.02	-0.55
	BapHdef-B5	11	-0.05	0.06	0.15	-0.75	-0.33	-0.14	-0.40	0.04	0.14	0.04	-0.14
	BapHdef-B1	82	-0.54	0.54	0.13	0.46	0.01	-0.12	0.38	0.12	-0.71	-0.18	0.71
	BapHdef-B4	42	0.73	-0.74	-0.24	-0.28	0.20	0.24	-0.26	-0.19	0.85	0.19	-0.85

Additional File1, Supplementary Figure 3 – Correlation of *B. aphidicola* and *H. defensa* gene modules. Pearson correlation coefficient between eigengenes of *B. aphidicola* and *H. defensa* modules. Coloured: Correlation has a p-value of <0.01, red indicates positive and blue indicates negative correlation.

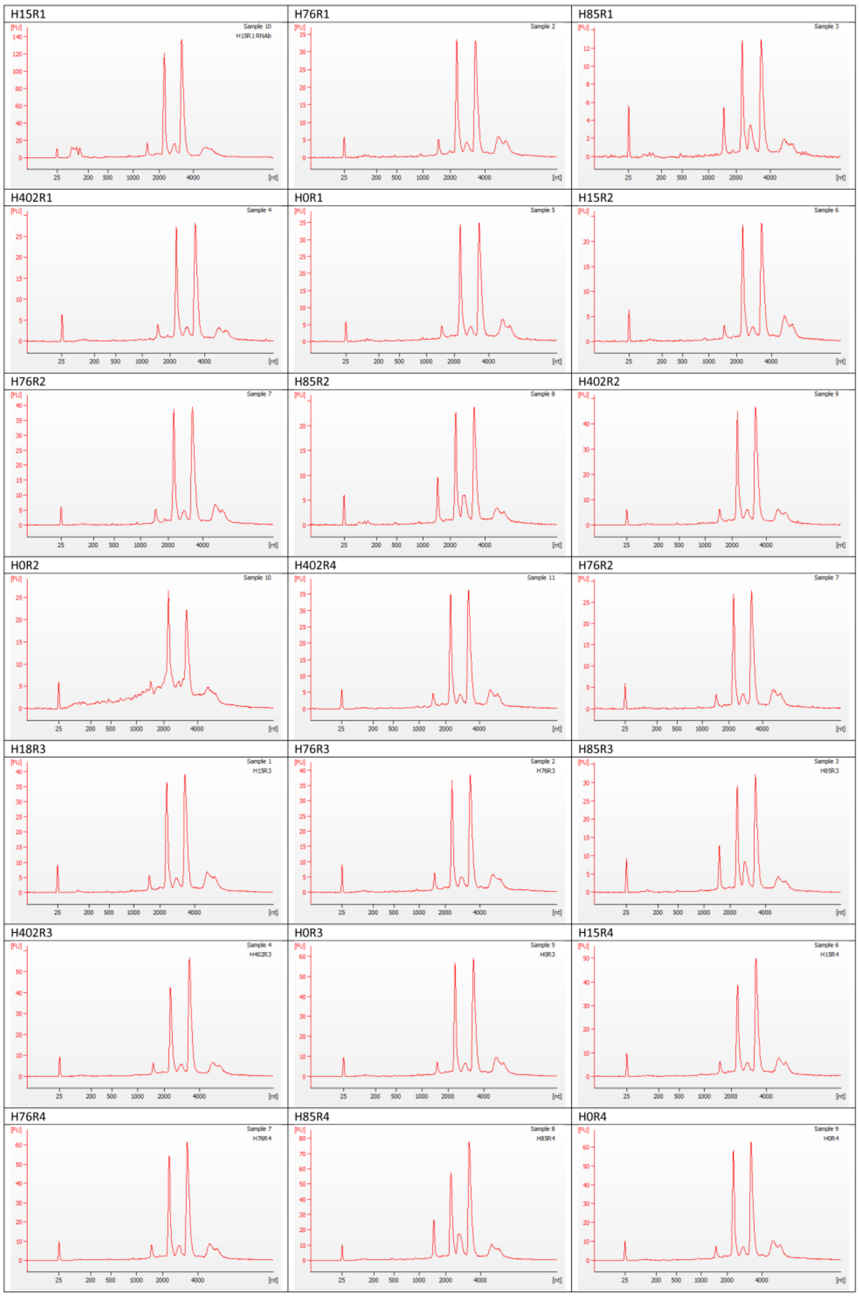

Additional File1, Supplementary Figure 4 – High RNA integrity after total RNA extraction. Bioanalyzer 2100 electopherograms of all libraries in batch A and B. Comparison with Schroeder et al. (2006) implied a RIN of 8 for sample H0R2 and 9- 10 for all others. Ribosomal RNA peaks of the different organisms are visible. Consider that a double 18S rRNA peak is expected for several insect species.
