# Supplementary material for: Triple RNA-Seq characterizes aphid gene expression in response to infection with unequally virulent strains of the endosymbiont Hamiltonella defensa
Source: BMC Genomics. 2021 Jun 16;22:449. doi: 10.1186/s12864-021-07742-8 (PMC8207614; doi:10.1186/s12864-021-07742-8)
Supplement: Supplementary file 4 — Additional File 4. R-scripts of the correlation approach and WGCNA analysis adapted from Smith et al. [51]. [file 12864_2021_7742_MOESM4_ESM.docx]

# R script for WGCNA Network Construction

# written by Thomas E. Smith (last uptdate in Sep 2019), for the publication:

# Smith TE, Moran NA. 2020. Coordination of host and symbiont gene expression reveals a metabolic tug-of-war

# between aphids and Buchnera. Proceedings of the National Academy of Sciences. 117(4):2113.

# adapted by Heidi Kaech (January 2021)

# Based on instructions in https://horvath.genetics.ucla.edu/html/CoexpressionNetwork/Rpackages/WGCNA/Tutorials/FemaleLiver-01-dataInput.pdf

# setting the stage

#wkdir <- "YOUR WORK DIRECTORY"

setwd(wkdir)

library("WGCNA")

library("flashClust")

library("ape")

library("ggtree")

library("RColorBrewer")

library("pheatmap")

options(stringsAsFactors = FALSE)

# load log-transformed counts from DESeq2

# must be read from .csv file, rld is an incompatible object type

# Aphid data

ApData <- read.table("FILE.csv", sep = ",", header=T, stringsAsFactors = F)

datExprAp0 <- as.data.frame(t(ApData[, -1]))

names(datExprAp0) <- ApData$X

rownames(datExprAp0) <- names(ApData[, -1])

# Hamiltonella data

HaData <- read.table("FILE.csv", sep = ",", header=T, stringsAsFactors = F)

datExprHa0 <- as.data.frame(t(HaData[, -1]))

names(datExprHa0) <- HaData$X

rownames(datExprHa0) <- names(HaData[, -1])

# check for columns with excessive missing values ("NA"s) and identify outliers

# For Aphid

gsg <- goodSamplesGenes(datExprAp0, verbose=3)

gsg$allOK

if (!gsg$allOK) {

# Optionally, print the gene and sample names that were removed:

if (sum(!gsg$goodGenes)>0)

printFlush(paste("Removing genes:", paste(names(datExprAp0)[!gsg$goodGenes], collapse = ", ")));

if (sum(!gsg$goodSamples)>0)

printFlush(paste("Removing samples:", paste(rownames(datExprAp0)[!gsg$goodSamples],

collapse = ", ")));

# Remove the offending genes and samples from the data:

datExprAp0 = datExprAp0[gsg$goodSamples, gsg$goodGenes]

}

# For Hamiltonella

gsg <- goodSamplesGenes(datExprHa0, verbose=3)

gsg$allOK

if (!gsg$allOK) {

if (sum(!gsg$goodGenes)>0)

printFlush(paste("Removing genes:", paste(names(datExprHa0)[!gsg$goodGenes], collapse = ", ")));

if (sum(!gsg$goodSamples)>0)

printFlush(paste("Removing samples:", paste(rownames(datExprHa0)[!gsg$goodSamples],

collapse = ", ")));

datExprHa0 = datExprHa0[gsg$goodSamples, gsg$goodGenes]

}

# cluster samples by expression

# For aphid

samp <- "Ap"

sampleTreeAp <- hclust(dist(datExprAp0), method = "average")

dev.off()

pdf(paste0("sampleTree_rld_", samp, ".pdf"), width=12, height=9)

par(cex=0.6)

par(mar=c(0,4,2,0))

plot(sampleTreeAp, main = "Sample clustering to detect outliers", sub="", xlab="",

cex.lab = 1.5, cex.axis = 1.5, cex.main = 2)

# look at pdf, choose a height to remove any outliers, and enter this value as cutHeightAp

# replot with final cutHeightAp value

cutHeightAp <- 13

abline(h=cutHeightAp, col="red")

dev.off()

# cut the tree to remove the outlier if necessary

# clustAp <- cutreeStatic(sampleTreeAp, cutHeight = cutHeightAp, minSize = 10)

# table(clustAp)

# # shows the clusters formed, clust 1 contains the desired samples

# keepSamples <- (clustAp==1)

datExprAp <- datExprAp0

nGenesAp <- ncol(datExprAp)

nSamplesAp <- nrow(datExprAp)

# For Hamiltonella

samp <- "Ha"

sampleTreeHa <- hclust(dist(datExprHa0), method = "average")

dev.off()

pdf(paste0("sampleTree_rld_", samp, ".pdf"), width=12, height=9)

par(cex=0.6)

par(mar=c(0,4,2,0))

plot(sampleTreeHa, main = "Sample clustering to detect outliers", sub="", xlab="",

cex.lab = 1.5, cex.axis = 1.5, cex.main = 2)

# look at pdf, choose a height to remove any outliers, and enter this

# value as cutHeightAp

# replot with final cutHeightAp value

cutHeightHa <- 20

abline(h=cutHeightHa, col="red")

dev.off()

# cut the tree to remove the outlier, if necessary

# clustHa <- cutreeStatic(sampleTreeHa, cutHeight = cutHeightHa, minSize = 10)

# table(clustHa)

# # shows the clusters formed, clust 1 contains the desired samples

# keepSamples <- (clustHa==1)

datExprHa <- datExprHa0

nGenesHa <- ncol(datExprHa)

nSamplesHa <- nrow(datExprHa)

# format trait data for WGCNA

sampleColor <- c("light blue", "light blue", "light blue", "orange", "orange", "orange",

"orange", "light grey", "light grey", "light grey", "light grey", "red",

"red", "red", "red")

sampleTiter <- c(1.06,1.44,0.76,2.13,2.05,2.50,2.32,2.15,2.35,

1.89,2.01,26.16,26.39,23.73,21.53)

# For aphid

names(sampleTiter) <- names(ApData[, 2:16])

names(sampleColor) <- names(ApData[, 2:16])

datTraitsAp0 <- as.data.frame(cbind(sampleTiter))

keepColumns <- (rownames(datTraitsAp0) %in% rownames(datExprAp))

datTraitsAp <- as.data.frame(datTraitsAp0[keepColumns,])

colnames(datTraitsAp) <- colnames(datTraitsAp0)

# For Hamiltonella

names(sampleTiter) <- names(HaData[, 2:16])

names(sampleColor) <- names(HaData[, 2:16])

datTraitsHa0 <- as.data.frame(cbind(sampleTiter))

keepColumns <- (rownames(datTraitsHa0) %in% rownames(datExprHa))

datTraitsHa <- as.data.frame(datTraitsHa0[keepColumns,])

colnames(datTraitsHa) <- colnames(datTraitsHa0)

# cluster samples again with outlier sample removed

# For Aphid

samp <- "Ap"

sampleTreeAp2_average <- hclust(dist(datExprAp), method = "average")

sampleTreeAp2<-sampleTreeAp2_average

# For Hamiltonella

samp <- "Ha"

sampleTreeHa2_average <- hclust(dist(datExprHa), method = "average")

sampleTreeHa2<-sampleTreeHa2_average

# For Aphid

samp <- "Ap"

titerColors <- numbers2colors(as.numeric(datTraitsAp$sampleTiter), signed=FALSE)

traitColors <- as.matrix(cbind(titerColors))

dev.off()

pdf(paste0("sampleTree2andTiter_rld", samp, ".pdf"), width=12, height=9)

plotDendroAndColors(sampleTreeAp2, traitColors,

groupLabels = names(datTraitsAp),

main = "Sample dendogram and trait heatmap")

dev.off()

# For Hamiltonella

samp <- "Ha"

titerColors <- numbers2colors(as.numeric(datTraitsHa$sampleTiter), signed=FALSE)

traitColors <- as.matrix(cbind(titerColors))

dev.off()

pdf(paste0("sampleTree2andTiter_rld_", samp, ".pdf"), width=12, height=9)

plotDendroAndColors(sampleTreeHa2, traitColors,

groupLabels = names(datTraitsHa),

main = "Sample dendogram and trait heatmap")

dev.off()

# selection of soft-thresholiding power by analysis of expression network fit

# with scale-free topology model; make sure genes are all processed in the same block.

# For Aphid

samp <- "Ap"

powers = c(c(1:10), seq(from = 12, to=20, by=2))

sft = pickSoftThreshold(datExprAp, powerVector = powers, verbose = 5, blockSize=10000,

networkType="signed hybrid", corFnc=bicor)

dev.off()

pdf(paste0("softThresholdPlots_rld_", samp, ".pdf"), width=12, height=9)

sizeGrWindow(12, 9)

par(mfrow = c(1,2))

cex1 = 0.8

plot(sft$fitIndices[,1], -sign(sft$fitIndices[,3])*sft$fitIndices[,2],

xlab="Soft Threshold (power)",ylab="Scale Free Topology Model Fit,signed R^2",

type="n", main = paste("Scale independence"))

text(sft$fitIndices[,1], -sign(sft$fitIndices[,3])*sft$fitIndices[,2],

labels=powers,cex=cex1,col="red")

# the next line adds a line at an R^2 cutoff value of 0.9

abline(h=0.9,col="blue")

plot(sft$fitIndices[,1], sft$fitIndices[,5],

xlab="Soft Threshold (power)",ylab="Mean Connectivity", type="n",

main = paste("Mean connectivity"))

text(sft$fitIndices[,1], sft$fitIndices[,5], labels=powers, cex=cex1,col="red")

dev.off()

# For Hamiltonella

samp <- "Ha"

powers = c(c(1:10), seq(from = 12, to=20, by=2))

sft = pickSoftThreshold(datExprHa, powerVector = powers, verbose = 5,

networkType="signed hybrid", corFnc=bicor)

dev.off()

pdf(paste0("softThresholdPlots_rld_", samp, ".pdf"), width=12, height=9)

sizeGrWindow(12, 9)

par(mfrow = c(1,2))

cex1 = 0.8

plot(sft$fitIndices[,1], -sign(sft$fitIndices[,3])*sft$fitIndices[,2],

xlab="Soft Threshold (power)",ylab="Scale Free Topology Model Fit,signed R^2",

type="n", main = paste("Scale independence"))

text(sft$fitIndices[,1], -sign(sft$fitIndices[,3])*sft$fitIndices[,2],

labels=powers,cex=cex1,col="red")

# the next line adds a line at an R^2 cutoff value of 0.8

abline(h=0.85,col="blue")

plot(sft$fitIndices[,1], sft$fitIndices[,5],

xlab="Soft Threshold (power)",ylab="Mean Connectivity", type="n",

main = paste("Mean connectivity"))

text(sft$fitIndices[,1], sft$fitIndices[,5], labels=powers, cex=cex1,col="red")

dev.off()

# look at 'softThresholdPlots.pdf' and decide on the smallest power level that

# gets above R^2 = 0.85 for Ha and 0.9 for Aphid

# set soft thresholding power and calculate adjacencies

softPowerAp <- 8

adjacencyAp <- adjacency(datExprAp, power = softPowerAp, type="signed hybrid",

corFnc = bicor)

softPowerHa <- 9

adjacencyHa <- adjacency(datExprHa, power = softPowerHa, type="signed hybrid",

corFnc = bicor)

# transform adjacencies into a Topological Overlap Matrix, calculate dissimilarity

TOMAp = TOMsimilarity(adjacencyAp, TOMType="signed")

dissTOMAp = 1-TOMAp

TOMHa = TOMsimilarity(adjacencyHa, TOMType="signed")

dissTOMHa = 1-TOMHa

# clustering using TOM

geneTreeAp = hclust(as.dist(dissTOMAp), method = "average")

geneTreeHa = hclust(as.dist(dissTOMHa), method = "average")

# plot the resulting gene clustering dendrograms

# For Aphid

samp <- "Ap"

dev.off()

pdf(paste0("geneTree_rld_", samp, ".pdf"), width=12, height=9)

sizeGrWindow(12,9)

print(plot(geneTreeAp, xlab="", sub="",

main = "Gene clustering on TOM-Hased dissimilarity, power level = 8",

labels = FALSE, hang = 0.04))

dev.off()

# For Hamiltonella

samp <- "Ha"

dev.off()

pdf(paste0("geneTree_rld_", samp, ".pdf"), width=12, height=9)

sizeGrWindow(12,9)

print(plot(geneTreeHa, xlab="", sub="",

main = "Gene clustering on TOM-Hased dissimilarity, power level = 9",

labels = FALSE, hang = 0.04))

dev.off()

# set the minimum module size relatively high

minModuleSizeAp = 20

minModuleSizeHa = 10

# module identification using dynamic tree cut:

dynamicModsAp = cutreeDynamic(dendro = geneTreeAp, distM = dissTOMAp,

deepSplit = 2, pamRespectsDendro = FALSE,

minClusterSize = minModuleSizeAp)

table(dynamicModsAp)

# for Hamiltonella

dynamicModsHa = cutreeDynamic(dendro = geneTreeHa, distM = dissTOMHa,

deepSplit = 2, pamRespectsDendro = FALSE,

minClusterSize = minModuleSizeHa)

table(dynamicModsHa)

# convert numeric lables into colors

dynamicColorsAp = labels2colors(dynamicModsAp)

table(dynamicColorsAp)

dynamicColorsHa = labels2colors(dynamicModsHa)

table(dynamicColorsHa)

# plot the dendrogram and colors underneath

# For Aphid

samp <- "Ap"

pdf(paste0("geneTreeModules_rld_", samp, ".pdf"), width=12, height=9)

sizeGrWindow(12,9)

print(plotDendroAndColors(geneTreeAp, dynamicColorsAp, "Dynamic Tree Cut",

dendroLabels = FALSE, hang = 0.03,

addGuide = TRUE, guideHang = 0.05,

main = "Gene dendrogram and module colors, power level = 10"))

dev.off()

# For Hamiltonella

samp <- "Ha"

dev.off()

pdf(paste0("geneTreeModules_rld_", samp, ".pdf"), width=12, height=9)

sizeGrWindow(12,9)

print(plotDendroAndColors(geneTreeHa, dynamicColorsHa, "Dynamic Tree Cut",

dendroLabels = FALSE, hang = 0.03,

addGuide = TRUE, guideHang = 0.05,

main = "Gene dendrogram and module colors, power level = 9"))

dev.off()

# merge modules with similar expression profiles

# calculate eigengenes

samp <- "Ap"

MEList = moduleEigengenes(datExprAp, colors = dynamicColorsAp)

MEsAp = MEList$eigengenes

# calculate dissimilarity of module eigengenes

MEDiss = 1-cor(MEsAp)

# cluster module eigengenes

METreeAp = hclust(as.dist(MEDiss), method = "average")

MEDissThresAp = 0.4

# plot results

# For Aphid

dev.off()

pdf(paste0("METree_rld_", samp, ".pdf"), width=12, height=9)

sizeGrWindow(12, 9)

print(plot(METreeAp, main = "Clustering of module eigengenes",

xlab = "", ylab="dissimilarity", sub = ""))

abline(h=MEDissThresAp, col = "red")

dev.off()

# look at METree.pdf and choose a cutoff height, below which modules will be merged

MEDissThresAp = 0.4

# For Hamiltonella

samp <- "Ha"

MEList = moduleEigengenes(datExprHa, colors = dynamicColorsHa)

MEsHa = MEList$eigengenes

# calculate dissimilarity of module eigengenes

MEDiss = 1-cor(MEsHa)

# cluster module eigengenes

METreeHa = hclust(as.dist(MEDiss), method = "average")

MEDissThresHa = 0.2

# plot results

dev.off()

pdf(paste0("METree_rld_", samp, ".pdf"), width=12, height=9)

sizeGrWindow(12, 9)

print(plot(METreeHa, main = "Clustering of module eigengenes",

xlab = "", ylab="dissimilarity", sub = ""))

abline(h=MEDissThresHa, col = "red")

dev.off()

# look at METree.pdf and choose a cutoff height, below which modules will be merged

MEDissThresHa = 0.2

# merge modules and assign new colors to merged modules

# For Aphid

mergeAp = mergeCloseModules(datExprAp, dynamicColorsAp, cutHeight = MEDissThresAp,

verbose = 3)

mergedColorsAp = mergeAp$colors

# eigengenes of the new merged modules:

mergedMEsAp = mergeAp$newMEs

# For Hamiltonella

mergeHa = mergeCloseModules(datExprHa, dynamicColorsHa, cutHeight = MEDissThresHa,

verbose = 3)

mergedColorsHa = mergeHa$colors

mergedMEsHa = mergeHa$newMEs

# re-plot results with merged modules

# For Aphid

samp <- "Ap"

dev.off()

pdf(paste0("geneTreeMergedModules_rld_", samp, ".pdf"), width=12, height=9)

sizeGrWindow(12, 9)

plotDendroAndColors(geneTreeAp, cbind(dynamicColorsAp, mergedColorsAp),

c("Dynamic Tree Cut", "Merged dynamic"),

dendroLabels = FALSE, hang = 0.03,

addGuide = TRUE, guideHang = 0.05)

dev.off()

# For Hamiltonella

samp <- "Ha"

dev.off()

pdf(paste0("geneTreeMergedModules_rld_", samp, ".pdf"), width=12, height=9)

sizeGrWindow(12, 9)

plotDendroAndColors(geneTreeHa, cbind(dynamicColorsHa, mergedColorsHa),

c("Dynamic Tree Cut", "Merged dynamic"),

dendroLabels = FALSE, hang = 0.03,

addGuide = TRUE, guideHang = 0.05)

dev.off()

# rename objects for next step

moduleColorsAp = mergedColorsAp

moduleColorsHa = mergedColorsHa

# construct numerical labels corresponding to the colors

colorOrder = c("grey", standardColors(50))

moduleLabelsAp = match(moduleColorsAp, colorOrder)-1

moduleLabelsHa = match(moduleColorsHa, colorOrder)-1

MEsAp = mergedMEsAp

MEsHa = mergedMEsHa

nGenesAp = ncol(datExprAp)

nSamplesAp = nrow(datExprAp)

# recalculate MEs with color label

# For Aphid

MEsAp = moduleEigengenes(datExprAp, moduleColorsAp)$eigengenes

MEsAp = orderMEs(MEsAp)

moduleTraitCorAp = cor(MEsAp, datTraitsAp, use = "p")

moduleTraitPvalueAp = corPvalueStudent(moduleTraitCorAp, nSamplesAp)

# For Hamiltonella

nGenesHa = ncol(datExprHa)

nSamplesHa = nrow(datExprHa)

MEsHa = moduleEigengenes(datExprHa, moduleColorsHa)$eigengenes

MEsHa = orderMEs(MEsHa)

moduleTraitCorHa = cor(MEsHa, datTraitsHa, use = "p")

moduleTraitPvalueHa = corPvalueStudent(moduleTraitCorHa, nSamplesHa)

# display correlations and their p-values in a heat map

# For Aphid

samp <- "Ap"

dev.off()

pdf(paste0("ModuleTraitCor_rld", samp, ".pdf"), width=12, height=9)

sizeGrWindow(12,9)

textMatrix = paste(signif(moduleTraitCorAp, 2), "\n(",

signif(moduleTraitPvalueAp, 1), ")", sep = "");

dim(textMatrix) = dim(moduleTraitCorAp)

par(mar = c(6, 8.5, 3, 3))

labeledHeatmap(Matrix = moduleTraitCorAp,

xLabels = names(datTraitsAp),

yLabels = names(MEsAp),

ySymbols = names(MEsAp),

colorLabels = FALSE,

colors = blueWhiteRed(50),

textMatrix = textMatrix,

setStdMargins = FALSE,

cex.text = 0.5,

zlim = c(-1,1),

main = paste("Module-trait relationships, power level 8"))

dev.off()

# For Hamiltonella

samp <- "Ha"

pdf(paste0("ModuleTraitCor_rld", samp, ".pdf"), width=12, height=9)

sizeGrWindow(12,9)

textMatrix = paste(signif(moduleTraitCorHa, 2), "\n(",

signif(moduleTraitPvalueHa, 1), ")", sep = "");

dim(textMatrix) = dim(moduleTraitCorHa)

par(mar = c(6, 8.5, 3, 3))

labeledHeatmap(Matrix = moduleTraitCorHa,

xLabels = names(datTraitsHa),

yLabels = names(MEsHa),

ySymbols = names(MEsHa),

colorLabels = FALSE,

colors = blueWhiteRed(50),

textMatrix = textMatrix,

setStdMargins = FALSE,

cex.text = 0.5,

zlim = c(-1,1),

main = paste("Module-trait relationships, power level 9"))

dev.off()

# calculate gene significance and module membership

# For Aphid

modNamesAp = substring(names(MEsAp), 3)

geneModuleMembershipAp = as.data.frame(cor(datExprAp, MEsAp, use = "p"))

MMPvalueAp = as.data.frame(corPvalueStudent(as.matrix(geneModuleMembershipAp),

nSamplesAp))

names(geneModuleMembershipAp) = paste("MM", modNamesAp, sep="")

names(MMPvalueAp) = paste("p.MM", modNamesAp, sep="")

geneTraitSignificanceAp = as.data.frame(cor(datExprAp, datTraitsAp, use = "p"))

GSPvalueAp = as.data.frame(corPvalueStudent(as.matrix(geneTraitSignificanceAp),

nSamplesAp))

names(geneTraitSignificanceAp) = paste("GS.", names(datTraitsAp), sep="")

names(GSPvalueAp) = paste("p.GS.", names(datTraitsAp), sep="")

# For Hamiltonella

modNamesHa = substring(names(MEsHa), 3)

geneModuleMembershipHa = as.data.frame(cor(datExprHa, MEsHa, use = "p"))

MMPvalueHa = as.data.frame(corPvalueStudent(as.matrix(geneModuleMembershipHa),

nSamplesHa))

names(geneModuleMembershipHa) = paste("MM", modNamesHa, sep="")

names(MMPvalueHa) = paste("p.MM", modNamesHa, sep="")

geneTraitSignificanceHa = as.data.frame(cor(datExprHa, datTraitsHa, use = "p"))

GSPvalueHa = as.data.frame(corPvalueStudent(as.matrix(geneTraitSignificanceHa),

nSamplesHa))

names(geneTraitSignificanceHa) = paste("GS.", names(datTraitsHa), sep="")

names(GSPvalueHa) = paste("p.GS.", names(datTraitsHa), sep="")

# identify genes with high significance (GS) for titer and high module membership (MM)

# for a module of interest

# "genes highly significantly associated with a trait are often also the most

# important (central) elements of modules associated with the trait"

# using module "darkturquoise" as an example for A. fabae

module = "darkolivegreen4"

column = match(module, modNamesAp)

moduleGenesAp = (moduleColorsAp==module)

# plot MM vs. GS for genes from this module

samp <- "Ap"

pdf(paste0("MMvGS_", module, "_rld", samp, ".pdf"), width=7, height=7)

sizeGrWindow(7, 7);

par(mfrow = c(1,1));

verboseScatterplot(abs(geneModuleMembershipAp[moduleGenesAp, column]),

abs(geneTraitSignificanceAp[moduleGenesAp, 1]),

xlab = paste("Module Membership in", module, "module"),

ylab = "Gene significance for titer",

main = paste("Module membership vs. gene significance\n"),

cex.main = 1.2, cex.lab = 1.2, cex.axis = 1.2, col = module)

dev.off()

pdf(paste0("MM_hist_", module, "_rld", samp, ".pdf"), width=7, height=7)

hist(abs(geneModuleMembershipAp[moduleGenesAp, column]),

breaks = 20, xlim = c(0, 1), ylim = c(0, 250),

xlab = paste("Module Membership"),

main = paste("Module Membership distribution in", module, "module"))

dev.off()

pdf(paste0("GS_hist_", module, "_rld", samp, ".pdf"), width=7, height=7)

hist(abs(geneTraitSignificanceAp[moduleGenesAp, 1]),

breaks = 20, xlim = c(0, 1), ylim = c(0, 200),

xlab = paste("Gene Significance for titer"),

main = paste("Gene Significance for titer distribution in", module, "module"))

dev.off()

# now for Hamiltonella, using most significantly correlated module

module = "salmon"

column = match(module, modNamesHa)

moduleGenesHa = (moduleColorsHa==module)

samp <- "Ha"

pdf(paste0("MMvGS_", module, "_rld", samp, ".pdf"), width=7, height=7)

sizeGrWindow(7, 7);

par(mfrow = c(1,1));

verboseScatterplot(abs(geneModuleMembershipHa[moduleGenesHa, column]),

abs(geneTraitSignificanceHa[moduleGenesHa, 1]),

xlab = paste("Module Membership in", module, "module"),

ylab = "Gene significance for Titer",

main = paste("Module membership vs. gene significance\n"),

cex.main = 1.2, cex.lab = 1.2, cex.axis = 1.2, col = module)

dev.off()

pdf(paste0("MM_hist_", module, "_rld", samp, ".pdf"), width=7, height=7)

hist(abs(geneModuleMembershipHa[moduleGenesHa, column]),

breaks = 20, xlim = c(0, 1), ylim = c(0, 8),

xlab = paste("Module Membership"),

main = paste("Module Membership distribution in", module, "module"))

dev.off()

pdf(paste0("GS_hist_", module, "_rld", samp, ".pdf"), width=7, height=7)

hist(abs(geneTraitSignificanceHa[moduleGenesHa, 1]),

breaks = 20, xlim = c(0, 1), ylim = c(0, 8),

xlab = paste("Gene Significance for Titer"),

main = paste("Gene Significance for Titer distribution in", module, "module"))

dev.off()

# prepare summary tables of results

# load deseq2 summary tables

resOrderedAp <- read.csv("resOrderedAp.csv",

quote = "\"", header = T, stringsAsFactors = F)

rownames(resOrderedAp) <- resOrderedAp[,1 ]

resOrderedAp <- resOrderedAp[,-1 ]

resOrderedHa <- read.csv("resOrderedHA.csv",

quote = "\"", header = T, stringsAsFactors = F)

rownames(resOrderedHa) <- resOrderedHa[,1 ]

resOrderedHa <- resOrderedHa[,-1]

# obtain the gene order from the datExpr objects

geneOrderAp <- colnames(datExprAp)

geneOrderHa <- colnames(datExprHa)

# set up tables combining WGCNA results with deseq2 results

resOrderedAp <- resOrderedAp[match(geneOrderAp, rownames(resOrderedAp)), ]

geneInfoAp0 <- data.frame(resOrderedAp, moduleColor = moduleColorsAp,

geneTraitSignificanceAp, GSPvalueAp)

resOrderedHa <- resOrderedHa[match(geneOrderHa, rownames(resOrderedHa)), ]

geneInfoHa0 <- data.frame(resOrderedHa, moduleColor = moduleColorsHa,

geneTraitSignificanceHa, GSPvalueHa)

# order modules by their significance for titer

sampleTiterAp <- datTraitsAp$sampleTiter

modOrderAp = order(-abs(cor(MEsAp, sampleTiterAp, use = "p")))

# and order Hamiltonella modules by Titer

sampleTiterHa <- datTraitsHa$sampleTiter

modOrderHa = order(-abs(cor(MEsHa, sampleTiterHa, use = "p")))

# add module membership information in the chosen order

# For Aphid

for (mod in 1:ncol(geneModuleMembershipAp)) {

oldNames = names(geneInfoAp0)

geneInfoAp0 = data.frame(geneInfoAp0, geneModuleMembershipAp[, modOrderAp[mod]],

MMPvalueAp[, modOrderAp[mod]])

names(geneInfoAp0) = c(oldNames, paste("MM.", modNamesAp[modOrderAp[mod]], sep=""),

paste("p.MM.", modNamesAp[modOrderAp[mod]], sep=""))

}

# For Hamiltonella

for (mod in 1:ncol(geneModuleMembershipHa)) {

oldNames = names(geneInfoHa0)

geneInfoHa0 = data.frame(geneInfoHa0, geneModuleMembershipHa[, modOrderHa[mod]],

MMPvalueHa[, modOrderHa[mod]])

names(geneInfoHa0) = c(oldNames, paste("MM.", modNamesHa[modOrderHa[mod]], sep=""),

paste("p.MM.", modNamesHa[modOrderHa[mod]], sep=""))

}

# order the genes in the geneInfo variable by deseq2 adj p-value, as in DESeq2 results table

geneOrderAp = order(geneInfoAp0$padj, decreasing = F)

geneOrderHa = order(geneInfoHa0$padj, decreasing = F)

# rename tables, keeping them in order of deseq2 results

WGCNAresAp = geneInfoAp0[geneOrderAp, ]

WGCNAresHa = geneInfoHa0[geneOrderHa, ]

# save as .csv files

write.table(WGCNAresAp, file = paste0("WGCNAresAp.txt"),

row.names = TRUE, quote=F, sep="\t")

write.table(WGCNAresHa, file = paste0("WGCNAresHa.txt"),

row.names = TRUE, quote=F, sep="\t")

#plot lists with module membership for GO enrichment analysis

# prepare an empty list to place Aphid gene names in

intModulesList.Ap <- list()

named.modlist.Ap<-moduleColorsAp

names(named.modlist.Ap)<-colnames(datExprAp)

# pick out gene names for every gene in each module of interest

for (module in unique(named.modlist.Ap)) {

# select module probes

modGenes = (named.modlist.Ap==module)

# get their gene names

modGenes = names(named.modlist.Ap)[modGenes]

# save objects in a list

intModulesList.Ap[[module]] <- modGenes

}

#write lists

for(item in unique(named.modlist.Ap)){

table<-as.data.frame(cbind(names(named.modlist.Ap),

ifelse(names(named.modlist.Ap)%in%intModulesList.Ap[[item]], 1, 0)))

write.table(file=paste("WGCNA_Ap_module_",item,".txt", sep=""),

quote=F, row.names = F, col.names=F,sep="\t", table)}

#plot lists with module membership for GO enrichment analysis

# prepare an empty list to place Hamiltonella gene names in

intModulesList.Ha <- list()

named.modlist.Ha<-moduleColorsHa

names(named.modlist.Ha)<-colnames(datExprHa)

# pick out gene names for every gene in each module of interest

for (module in unique(named.modlist.Ha)) {

# select module probes

modGenes = (named.modlist.Ha==module)

# get their gene names

modGenes = names(named.modlist.Ha)[modGenes]

# save objects in a list

intModulesList.Ha[[module]] <- modGenes

}

#write lists

for(item in unique(named.modlist.Ha)){

table<-as.data.frame(cbind(names(named.modlist.Ha),

ifelse(names(named.modlist.Ha)%in%intModulesList.Ha[[item]], 1, 0)))

write.table(file=paste("WGCNA_Ha_module_",item,".txt", sep=""),

quote=F, row.names = F, col.names=F,sep="\t", table)}

##################################################################################

# R script for correlation analysis of host and symbiont DESeq2 log-transformed counts

# written by Thomas E. Smith 2019 for pulication:

#Smith TE, Moran NA. 2020. Coordination of host and symbiont gene expression reveals a metabolic tug-of-war

# between aphids and Buchnera. Proceedings of the National Academy of Sciences. 117(4):2113.

# updated by Heidi Kaech January 2021

# setting the stage

wkdir <- "YOUR WORK DIRECTORY" ##adapt

setwd(wkdir)

## correlation of aphid and Hamiltonella genes

## identify and remove outlier samples from dataset (from WGCNA tutorial)

library("WGCNA")

library("flashClust")

##Start with Aphid data

# load rld data

samp <- "Ap"

rldAp00 <- read.table("rldAp.csv", sep = ",", header=T,

stringsAsFactors = F) ##(Replace this with Buchnera file for Correlating Bu and Ha)

rownames(rldAp00) <- rldAp00[,1]

rldAp00[,1] <- NULL

datExprAp <- as.data.frame(t(rldAp00))

# cluster samples with similar patterns of gene expression

# look at resulting pdf, choose a height to remove any outliers, and enter this value as cutHeightAp

# then replot with final cutHeightAp value

sampleTreeAp <- hclust(dist(datExprAp), method = "average")

pdf(paste0("sampleTree_rld", samp, ".pdf"), width=12, height=9)

par(cex=0.6)

par(mar=c(0,4,2,0))

plot(sampleTreeAp, main = "Sample clustering to detect outliers", sub="",

xlab="", cex.lab = 1.5, cex.axis = 1.5, cex.main = 2)

cutHeightAp <- 12 ##(Replace this with 3 when working with Buchnera)

abline(h=cutHeightAp, col="red")

dev.off()

# which sample was/were outlier(s)?

outlierAp <- "NA"

## do the same thing for Hamiltonella

# load rld data

samp <- "Ha"

rldHa00 <- read.table("rldHA.csv", sep = ",", header=T,

stringsAsFactors = F)

rownames(rldHa00) <- rldHa00[,1]

rldHa00[,1] <- NULL

datExprHa <- as.data.frame(t(rldHa00))

# cluster samples with similar patterns of gene expression

# look at resulting pdf, choose a height to remove any outliers, and enter this value as cutHeightHa

# then replot with final cutHeightHa value

sampleTreeHa <- hclust(dist(datExprHa), method = "average")

pdf(paste0("sampleTree_rld", samp, ".pdf"), width=12, height=9)

par(cex=0.6)

par(mar=c(0,4,2,0))

plot(sampleTreeHa, main = "Sample clustering to detect outliers", sub="",

xlab="", cex.lab = 1.5, cex.axis = 1.5, cex.main = 2)

cutHeightHa <- 19

abline(h=cutHeightHa, col="red")

dev.off()

# which sample was/were outlier(s)?

outlierHa <- "NA"

## rename both data sets

rldAp0 <- rldAp00

rldAp <- rldAp0

rldHa0 <- rldHa00

rldHa <- rldHa0

## calculate the inner quartile range (IQR) of each gene to use

# as a measure of variation among samples

samp <- "Ap"

# calculate the IQR for each gene based on rld data

rldApIqr <- apply(as.data.frame(rldAp), 1, IQR)

range(rldApIqr)

mean(rldApIqr)

pdf(paste0("IQR_rld", samp, ".pdf"), width=12, height=9)

hist(rldApIqr[rldApIqr < 2], xlim = c(0,2), breaks = 40, labels=T)

iqrCutoffAp <- 0.15 ##(Replace this with 0.1 when working with Buchnera)

abline(v=iqrCutoffAp, col="red")

dev.off()

IQRselectionAp <- rldApIqr > iqrCutoffAp

# what percentage of the total genes does this cutoff represent?

print(paste0("This represents ", ((sum(IQRselectionAp)/length(IQRselectionAp))*100),

" % of the total genes"))

## do the same for Hamiltonella

samp <- "Ha"

# calculate the IQR for each gene based on rld data

rldHaIqr <- apply(as.data.frame(rldHa), 1, IQR)

range(rldHaIqr)

mean(rldHaIqr)

pdf(paste0("IQR_rld", samp, ".pdf"), width=12, height=9)

hist(rldHaIqr[rldHaIqr < 2], xlim = c(0,2), breaks = 40, labels=T)

iqrCutoffHa <- 0.75

abline(v=iqrCutoffHa, col="red")

dev.off()

IQRselectionHa <- rldHaIqr > iqrCutoffHa

# what percentage of the total genes does this cutoff represent?

print(paste0("This represents ", ((sum(IQRselectionHa)/length(IQRselectionHa))*100),

" % of the total genes"))

## include in this analysis only those genes with padj < 0.05 from deseq2

## or exclude those with IQR below the IQR treshhold to remove genes that

## do not vary between samples or are not significant from the gene universe

# load deseq2 results table to obtain padj values

resAp <- read.table("resOrderedAp.csv",

sep = ",", header=T, stringsAsFactors = F)

rownames(resAp) <- resAp[,1]

resHa <- read.table("resOrderedHA.csv",

sep = ",", header=T, stringsAsFactors = F)

rownames(resHa) <- resHa[,1]

table(rownames(rldAp)%in%rownames(resAp))

resAp<-resAp[rownames(rldAp),]

table(rownames(rldAp)==rownames(resAp))

resHa<-resHa[rownames(rldHa),]

table(rownames(rldHa)==rownames(resHa))

## now remove genes that don't meet the above criteria

species <- c("Ap", "Ha")

rldList <- list(rldAp, rldHa)

resList <- list(resAp, resHa)

iqrList <- list(rldApIqr, rldHaIqr)

iqrCutoffList <- list(iqrCutoffAp, iqrCutoffHa)

for (i in 1:length(rldList)) {

rld <- rldList[[i]]

res <- resList[[i]]

rldIQR <- iqrList[[i]]

cutoff <- iqrCutoffList[[i]]

# ensure row order of res matches that of rld

res <- res[match(row.names(rld), rownames(res)), ]

# create empty data.frames to fill in

rldFilt <- data.frame()

resFilt <- data.frame()

if (nrow(res) == nrow(rld) & nrow(res) == length(rldIQR)) {

for (j in 1:nrow(rld)) {

if (!is.na(res$padj[j])) {

if (rldIQR[j] > cutoff | res$padj[j] < 0.01) {

rldFilt <- rbind(rldFilt, rld[j, ])

resFilt <- rbind(resFilt, res[j, ])

}

}

assign(paste0("rld", species[i], "Filt"), rldFilt)

assign(paste0("res", species[i], "Filt"), resFilt)

}

}

}

## create a matrix of correlations between counts of each pair of aphid and

## Hamiltonella genes. This will take a while...

c1 <- rldApFilt

c2 <- rldHaFilt

corMatrixNames <- list(row.names(c1), row.names(c2))

corMatrix <- matrix(nrow = nrow(c1), ncol = nrow(c2), dimnames = corMatrixNames)

for (i in 1:nrow(c2)) {

HaGene <- row.names(c2)[i]

for (j in 1:nrow(c1)) {

ApGene <- row.names(c1)[j]

countsComp <- data.frame(as.numeric(c1[ApGene,]), as.numeric(c2[HaGene,]))

countsCor <- cor(countsComp[,1], countsComp[,2], method = "pearson")

corMatrix[ApGene, HaGene] <- countsCor

}

}

nSamples <- ncol(rldApFilt)

corMatrixPval = corPvalueStudent(corMatrix, nSamples)

# check matrix validity with clustsig: columns (Hamiltonella)

library("clustsig")

colSimprof<-simprof(corMatrix, num.expected=100, num.simulated = 99, method.cluster="complete",

method.distance="euclidean", alpha=0.01,silent=F, sample.orientation="column",

increment=100)

colSimprofPlot<-simprof.plot(colSimprof)

dev.off()

pdf(paste0("Simprof_Ha.pdf"), width=100, height=30, pointsize=6)

simprof.plot(colSimprof, leafcolors = rep(c("purple","red","orange","yellow","lightgreen",

"green", "cyan","blue", "midnightblue","brown"),80))

dev.off()

# check matrix validity with clustsig: rows (aphid)

rowSimprof<-simprof(corMatrix, num.expected=100, num.simulated = 99, method.cluster="complete",

method.distance="euclidean", alpha=0.01,silent=F, sample.orientation="row", increment=100)

rowSimprofPlot<-simprof.plot(rowSimprof)

dev.off()

pdf(paste0("Simprof_Ap.pdf"), width=100, height=30, pointsize=6)

simprof.plot(rowSimprof, leafcolors = rep(c("purple","red","orange","yellow","lightgreen", "green",

"cyan","blue", "grey","brown"),80))

dev.off()

# look at the distribution of correlation coefficients in the matrix

pdf(paste0("ApHaCorrelations_corMatrix.pdf"), width=12, height=9)

hist(corMatrix, xlim = c(-1,1), ylim = c(0, 160000), breaks = 20, labels=T)

dev.off()

# look at the distribution of p-values in the matrix

pdf(paste0("ApHaCorrelationPvalues_corMatrixPval.pdf"), width=12, height=9)

hist(corMatrixPval, xlim = c(0,1), ylim = c(0, 500000), breaks = 20, labels=T)

dev.off()

## use cluster to determine optimal number of clusters

## pam/Gap works on columns

library(cluster)

pam1<-function(x,k) list(cluster=pam(x,k, cluster.only=T))

dev.off()

pdf("Gap_statistic_Ha.pdf")

pam.gap2<- clusGap(t(corMatrix), FUN=pam1, K.max=35, B=10)

plot(pam.gap2, main="Gap statistic for Hamiltonella data")

dev.off()

print(pam.gap2)

##Look at output to find 'good' number of groups

dev.off()

pdf("Gap_statistic_Ap.pdf")

pam.gap3<- clusGap((corMatrix), FUN=pam1, K.max=35, B=10)

plot(pam.gap3, main="Gap statistic for Aphid data")

dev.off()

##Look at output to find 'good' number of groups

# save the matrix so it can be accessed later

write.csv(as.data.frame(corMatrix),

file = paste0("GeneCorrelationMatrix_corMatrix_rld.csv"))

write.csv(as.data.frame(corMatrixPval),

file = paste0("GeneCorrelationMatrixPval_corMatrixPval_rld.csv"))

#### make a heatmap of the correlation matrix, with genes clustered by similar

#### patterns of gene correlation

## cluster rows and columns by similar correlation profiles

## first for Hamiltonella

library("stats")

library("flashClust")

library("WGCNA")

library("dendextend")

colDist <- dist(t(corMatrix))

colClust <- hclust(colDist)

dev.off()

pdf("Ha_cor_tree.pdf", width=30, height=15)

plot(colClust, main = "Ha correlation dendrogram", xlab = "", sub = "", cex=0.1 )

abline(h=18, col="red") ##(replace this with 6.25 when working with Buchnera)

dev.off()

# combine similar branches and group genes together: Hamiltonella

colClustCut <- cutree(colClust, h=18) ##(replace this with 6.25 when working with Buchnera)

table(colClustCut)

colClustCutCol <- labels2colors(colClustCut)

table(colClustCutCol)

names(colClustCutCol) <- colClust[["labels"]]

pdf("Ha_cor_tree_modules.pdf")

print(plotDendroAndColors(colClust, colClustCutCol, "Dynamic Tree Cut",

dendroLabels = FALSE, hang = 0.03,

addGuide = TRUE, guideHang = 0.05,

main = "Ha correlation dendrogram with clusters"))

dev.off()

## now clustering for the aphid

rowDist <- dist(corMatrix)

rowClust <- hclust(rowDist)

pdf("Ap_cor_tree.pdf")

plot(rowClust, main = "Ap correlation dendrogram", xlab = "", sub = "", cex=0.1)

abline(h=20, col="red") ##(Replace with 17 if working with Buchnera)

dev.off()

# combine similar branches and group genes together

rowClustCut <- cutree(rowClust, h=20)

table(rowClustCut)

rowClustCutCol <- labels2colors(rowClustCut)

table(rowClustCutCol)

names(rowClustCutCol) <- rowClust[["labels"]]

pdf("Ap_cor_tree_modules.pdf")

print(plotDendroAndColors(rowClust, rowClustCutCol, "Dynamic Tree Cut",

dendroLabels = FALSE, hang = 0.03,

addGuide = TRUE, guideHang = 0.05,

main = "Ap correlation dendrogram with clusters"))

dev.off()

## generate a heatmap for correlations between individual genes

# function to convert color name to hex code

col2hex <- function(color) {

df <- col2rgb(color)

hex <- rgb(df[1,], df[2,], df[3,], max = 255)

return(hex)

}

# set colors for heatmap

library("pheatmap")

# assign colors to each gene name for both Ap and Ha

rowAnnotation <- data.frame(ApModule = rowClustCutCol)

rownames(rowAnnotation) <- rowClust[["labels"]]

colAnnotation <- data.frame(HaModule = colClustCutCol)

rownames(colAnnotation) <- colClust[["labels"]]

# define color to be assigned to each module name and arrange it in a list

rowCol <- unique(rowClustCutCol) %>% col2hex()

names(rowCol) <- unique(rowClustCutCol)

colCol <- unique(colClustCutCol) %>% col2hex()

names(colCol) <- unique(colClustCutCol)

annotationColors <- list(ApModule = rowCol, HaModule = colCol)

# set up a diverging color scheme for correlation data so we can see both neg and pos correlations

library("RColorBrewer")

heatmapColors <- colorRampPalette(brewer.pal(8, "Spectral"))(20) %>% rev()

# order is blue to red (neg to pos)

# define legend breakpoints (for the 21 colors in heatmapColors)

breakList <- seq(-1, 1, by = 0.1)

# make the gene correlation heatmap using pretty heatmap package

# first save the heatmap as an object, to extract the gene order later

corMap <- pheatmap(corMatrix, color = heatmapColors, scale = "none",

main = "Gene correlation heatmap",

cutree_rows = length(rowCol), cutree_cols = length(colCol),

legend_breaks = breakList,

fontsize = 6, fontsize_row = 1, fontsize_col = 1,

annotation_row = rowAnnotation, annotation_col = colAnnotation,

annotation_names_row = F, annotation_names_col = F,

annotation_colors = annotationColors, annotation_legend = T,

breaks = breakList,

angle_col = "45")

dev.off()

# then save as image files. First, a pdf you can zoom in on to enable you to look

# at row/column labels

pdf(paste0("correlation_heatmap_ApHa.pdf"), width = 40, height = 60)

pheatmap(corMatrix, color = heatmapColors, scale = "none",

main = "Gene correlation heatmap",

cutree_rows = length(rowCol), cutree_cols = length(colCol),

legend_breaks = breakList,

fontsize = 6, fontsize_row = 1, fontsize_col = 1,

annotation_row = rowAnnotation, annotation_col = colAnnotation,

annotation_names_row = F, annotation_names_col = F,

annotation_colors = annotationColors, annotation_legend = T,

breaks = breakList,

angle_col = "45")

dev.off()

# then as a png for smaller image size and to use as an actual figure

png(paste0("correlation_heatmap_ApHa.png"), width = 800, height = 1000, res=100)

pheatmap(corMatrix, color = heatmapColors, scale = "none",

main = "Gene correlation heatmap",

cutree_rows = length(rowCol), cutree_cols = length(colCol),

legend_breaks = breakList,

fontsize = 10, fontsize_row = 0.1, fontsize_col = 0.1,

annotation_row = rowAnnotation, annotation_col = colAnnotation,

annotation_names_row = FALSE, annotation_names_col = FALSE,

annotation_colors = annotationColors, annotation_legend = T,

show_rownames=F, show_colnames=F,

breaks = breakList,

angle_col = "45")

dev.off()

## generate matrix figures for p-value matrix, too

# obtain gene order of corMap object

rowOrder <- corMap$tree_row[["order"]]

colOrder <- corMap$tree_col[["order"]]

# arrange p-value matrix accordingly

corMatrixPvalOrdered <- corMatrixPval[rowOrder, colOrder]

# new unidirectional color scheme and break numbers

heatmapColors <- colorRampPalette(brewer.pal(8, "YlOrRd"))(20) %>% rev()

# significant p-values are red

breakList <- seq(0, 1, by = 0.05)

# create vectors to specify where to introduce gaps between gene modules

# first, arrange gene colors in the same order as corMap

rowCCCordered <- rowClustCutCol[rowOrder]

colCCCordered <- colClustCutCol[colOrder]

# establish the order of module colors in corMap

rowColOrdered <- unique(rowCCCordered)

colColOrdered <- unique(colCCCordered)

# how many genes for each module color?

rowColCount <- table(rowCCCordered)[rowColOrdered]

colColCount <- table(colCCCordered)[colColOrdered]

# the vector to specify gap positions should add the number of genes in the next module to the

# number of genes in the previous

rowGap <- c(0)

for (i in 1:length(rowColCount)) {

previous <- rowGap[i]

current <- rowColCount[i] + previous

rowGap <- c(rowGap, current)

}

colGap <- c(0)

for (i in 1:length(colColCount)) {

previous <- colGap[i]

current <- colColCount[i] + previous

colGap <- c(colGap, current)

}

# heatmap object: unordered pvalues: CorMatrixPval.

corPvalMap <- pheatmap(corMatrixPval, color = heatmapColors, scale = "none",

main = "Gene correlation p-value heatmap",

cluster_cols = F, cluster_rows = F,

gaps_row = rowGap, gaps_col = colGap,

legend_breaks = breakList,

fontsize = 6, fontsize_row = 1, fontsize_col = 1,

annotation_row = rowAnnotation, annotation_col = colAnnotation,

annotation_names_row = F, annotation_names_col = F,

annotation_colors = annotationColors, annotation_legend = T,

breaks = breakList,

angle_col = "45")

# heatmap object: ordered pvalues: CorMatrixPvalOrdered.

dev.off()

png(paste0("correlation_heatmap_pval_ApHa.png"), width = 800, height = 1000,

res=100)

pheatmap(corMatrixPvalOrdered, color = heatmapColors, scale = "none",

main = "Gene correlation p-value heatmap (ordered)",

cluster_cols = F, cluster_rows = F,

gaps_row = rowGap, gaps_col = colGap,

legend_breaks = breakList,

fontsize = 10, fontsize_row = 0.1, fontsize_col = 0.1,

annotation_row = rowAnnotation, annotation_col = colAnnotation,

annotation_names_row = F, annotation_names_col = F,

annotation_colors = annotationColors, annotation_legend = T,

breaks = breakList,show_rownames=F, show_colnames=F,

angle_col = "45")

dev.off()

## create a correlation matrix of module eigengenes between Ap and Ha modules

# calculate module eigengenes for Ha

library("WGCNA")

MEListHa <- moduleEigengenes(t(rldHaFilt), colors = colClustCutCol)

MEsHa <- MEListHa$eigengenes

rownames(MEsHa) <- colnames(rldHaFilt)

# calculate module eignegenes for Ap

MEListAp <- moduleEigengenes(t(rldApFilt), colors = rowClustCutCol)

MEsAp <- MEListAp$eigengenes

rownames(MEsAp) <- colnames(rldApFilt)

# order the modules eigengenes to match the order of the gene correlation heatmap

rowGeneOrder <- rowClustCutCol[ corMap$tree_row[["order"]] ] # the order of Ap genes with module colors

rowColorOrder <- unique(rowGeneOrder) # the order of the Ap module colors

colGeneOrder <- colClustCutCol[ corMap$tree_col[["order"]] ] # the order of Ha genes with module colors

colColorOrder <- unique(colGeneOrder) # the order of the Ha module colors

MEsAp <- MEsAp[, match(rowColorOrder, gsub("ME", "", colnames(MEsAp)))]

MEsHa <- MEsHa[, match(colColorOrder, gsub("ME", "", colnames(MEsHa)))]

# calculate percentage of variability explained by each eigengene

MEsVarAp <- propVarExplained(t(rldApFilt), rowClustCutCol, MEsAp, corFnc = "cor",

corOptions = "use = 'p'")

MEsVarAp <- (MEsVarAp * 100)

MEsVarHa <- propVarExplained(t(rldHaFilt), colClustCutCol, MEsHa, corFnc = "cor",

corOptions = "use = 'p'")

MEsVarHa <- (MEsVarHa * 100)

# create eigengene correlation matrix

e1 <- t(MEsAp)

e2 <- t(MEsHa)

eMatrixNames <- list(row.names(e1), row.names(e2))

eMatrix <- matrix(nrow = nrow(e1), ncol = nrow(e2), dimnames = eMatrixNames)

for (i in 1:nrow(e2)) {

HaMod <- row.names(e2)[i]

for (j in 1:nrow(e1)) {

ApMod <- row.names(e1)[j]

eComp <- data.frame(as.numeric(e1[ApMod,]), as.numeric(e2[HaMod,]))

eCor <- cor(eComp[,1], eComp[,2], method = "pearson")

eMatrix[ApMod, HaMod] <- eCor

}

}

# calculate p-values for gene correlations

nSamples <- nrow(MEsAp)

eMatrixPval = corPvalueStudent(eMatrix, nSamples)

# set up annotation for heatmap

rowAnnotation <- data.frame(ApModule = rev(rowColorOrder))

rownames(rowAnnotation) <- rev(rownames(eMatrix))

colAnnotation <- data.frame(HaModule = colColorOrder)

rownames(colAnnotation) <- colnames(eMatrix)

# define color to be assigned to each module name and arrange it in a list

rowColors <- rowColorOrder

names(rowColors) <- rowColorOrder

colColors <- colColorOrder

names(colColors) <- colColorOrder

annotationColors <- list(ApModule = rowColors, HaModule = colColors)

## create a heatmap of eigengene correlation matrix

library("pheatmap")

library("RColorBrewer")

# establish colors to be used for heatmap

heatmapColors <- rev(colorRampPalette(brewer.pal(8, "Spectral"))(20))

# order is blue to red

# define legend breakpoints (for the 21 colors in heatmapColors)

breakList <- seq(-1, 1, by = 0.1)

dev.off()

png(paste0("eigengene_rlog_correlation_heatmap.png"), width = 800, height = 1000,

res=100)

pheatmap(eMatrix, color = heatmapColors, scale = "none",

main = "Eigengene correlation heatmap",

cluster_cols = F, cluster_rows = F,

legend_breaks = breakList,

fontsize = 10, fontsize_row = 10, fontsize_col = 10,

annotation_row = rowAnnotation, annotation_col = colAnnotation,

annotation_names_row = T, annotation_names_col = T,

annotation_colors = annotationColors, annotation_legend = F,

breaks = breakList,

angle_col = "45")

dev.off()

heatmapColors <- rev(colorRampPalette(brewer.pal(8, "Spectral"))(10))

# order is blue to red

# define legend breakpoints (for the 21 colors in heatmapColors)

breakList <- seq(0, 0.1, by = 0.01)

dev.off()

png(paste0("eigengene_rlog_correlation_pval_heatmap.png"), width = 800, height = 1000,

res=100)

pheatmap(eMatrixPval, color = heatmapColors, scale = "none",

main = "Eigengene correlation p-value heatmap",

cluster_cols = F, cluster_rows = F,

legend_breaks = breakList,

fontsize = 10, fontsize_row = 10, fontsize_col = 10,

annotation_row = rowAnnotation, annotation_col = colAnnotation,

annotation_names_row = T, annotation_names_col = T,

annotation_colors = annotationColors, annotation_legend = F,

breaks = breakList,

angle_col = "45")

dev.off()

## correlate global correlation analysis module eigengenes with titer

# calculate module membership of each gene

MMAp = as.data.frame(cor(t(rldApFilt), MEsAp, method = "pearson"))

MMPvalueAp <- corPvalueStudent(as.matrix(MMAp), nSamples = 19)

MMHa = as.data.frame(cor(t(rldHaFilt), MEsHa, method = "pearson"))

MMPvalueHa <- corPvalueStudent(as.matrix(MMHa), nSamples = 19)

# format sampleTiter to correlate titer with module eigengenes

sampleTiter <- c(1.06,1.44,0.76,2.13,2.05,2.50,2.32,2.15,2.35,1.89,2.01,26.16,26.39,23.73,21.53)

names(sampleTiter) <- colnames(rldApFilt)

# combine titer a single data.frame, with samples as rownames

datTraits <- data.frame(titer = sampleTiter)

# correlate eigengenes with titer, calculate p-values of correlation

ApModuleTiterCor <- cor(MEsAp, datTraits, use = "p")

ApModuleTiterCorP <- corPvalueStudent(ApModuleTiterCor, nSamples = 15)

# order MEsVar to match ApModuleTiterCor

MEsVarApOrdered <- MEsVarAp[match( gsub("ME", "", rownames(ApModuleTiterCor)),

gsub("PVE", "", names(MEsVarAp)) )]

ApModuleSummary <- data.frame(var.explained = MEsVarApOrdered,

cor.titer = ApModuleTiterCor[,1],

cor.titer.p.val = ApModuleTiterCorP[,1])

HaModuleTraitsCor <- cor(MEsHa, datTraits, use = "p")

HaModuleTraitsCorP <- corPvalueStudent(HaModuleTraitsCor, nSamples = 15)

# order MEsVar to match ApModuleTiterCor

MEsVapHaOrdered <- MEsVarHa[match( gsub("ME", "", rownames(HaModuleTraitsCor)),

gsub("PVE", "", names(MEsVarHa)) )]

HaModuleSummary <- data.frame(var.explained = MEsVapHaOrdered,

cor.titer = HaModuleTraitsCor[,1],

cor.titer.p.val = HaModuleTraitsCorP[,1])

# add correlation coefficients with other modules and p-values

for (mod in 1:ncol(eMatrix)) {

oldNames <- names(ApModuleSummary)

ApModuleSummary <- data.frame(ApModuleSummary,

eMatrix[ , mod],

eMatrixPval[ , mod])

names(ApModuleSummary) <- c(oldNames,

paste0("Ha.", gsub("ME", "", colnames(eMatrix))[mod]),

paste0("p.Ha.", gsub("ME", "", colnames(eMatrixPval))[mod]) )

}

rownames(ApModuleSummary) <- gsub("PVE", "Ap.", rownames(ApModuleSummary))

# and for Hamiltonella

for (mod in 1:nrow(eMatrix)) {

oldNames <- names(HaModuleSummary)

HaModuleSummary <- data.frame(HaModuleSummary,

eMatrix[mod, ],

eMatrixPval[mod, ])

names(HaModuleSummary) <- c(oldNames,

paste0("Ap.", gsub("ME", "", rownames(eMatrix))[mod]),

paste0("p.Ap.", gsub("ME", "", rownames(eMatrixPval))[mod]) )

}

rownames(HaModuleSummary) <- gsub("PVE", "Ha.", rownames(HaModuleSummary))

# save module summaries as tables

write.table(as.data.frame(ApModuleSummary),

file = paste0("ApModuleSummary_rld.txt"), quote=F, sep="\t")

write.table(as.data.frame(HaModuleSummary),

file = paste0("HaModuleSummary_rld.txt"), quote=F, sep="\t")

## plot Aphid module eigengenes against titer

library("ggplot2")

library("reshape")

# function to convert color name to hex code

col2hex <- function(color) {

df <- col2rgb(color)

hex <- rgb(df[1,], df[2,], df[3,], max = 255)

return(hex)

}

# plot relationship of eigengenes to titer or both A. pisum and Hamiltonella

species <- c("Ap", "Ha")

for (i in 1:length(species)) {

if (species[i] == "Ap") {

MEs <- MEsAp

}

if (species[i] == "Ha") {

MEs <- MEsHa

}

dat0 <- cbind(sampleTiter, MEs)

dat <- melt(dat0, id.vars = "sampleTiter", value.name = "RLD", variable = "Module")

dat$Module <- gsub("ME", "", dat$Module)

dat <- dat[order(dat$Module), ]

MEcol <- col2hex(unique(dat$Module))

# calculate mean rld counts for each module + titer level combination

meandat <- aggregate(dat$value, by = list(sampleTiter = dat$sampleTiter,

Module = dat$Module), FUN = mean)

# plot of module trendlines for titer, from linear model of eigengenes

line_plot0 <- ggplot(dat, aes(x = sampleTiter, y = value, color = Module)) +

geom_smooth(method = lm, se = F, size =0.5, show.legend = F) +

scale_color_manual(values = MEcol)+scale_x_log10()

line_plot <- line_plot0 + coord_cartesian(ylim = c(-0.4, 0.4)) +

theme(plot.title = element_blank(),

panel.background = element_rect(fill = "grey90"),

panel.grid.major = element_blank(),

panel.grid.minor = element_blank(),

axis.ticks = element_line(size = 2),

axis.ticks.length = unit(7, "pt"),

axis.line = element_line(color = "black"),

axis.text = element_text(color= "black", size = 20),

axis.title = element_blank() )

pdf(paste0("corr_analysis_ApHa/",species[i], "_EigengeneTrends_v_Titer.pdf"))

plot(line_plot)

dev.off()

# plot of module eigengenes for titer, with each point representing a mean of 2-3 replicates

line_plot0 <- ggplot(meandat, aes(x = sampleTiter, y = x, color = Module)) +

geom_line(size = 0.5, show.legend = F) +

scale_color_manual(values = MEcol)+scale_x_log10()

line_plot <- line_plot0 + coord_cartesian(ylim = c(-0.4, 0.4)) +

theme(plot.title = element_blank(),

panel.background = element_rect(fill = "grey90"),

panel.grid.major = element_blank(),

panel.grid.minor = element_blank(),

axis.ticks = element_line(size = 2),

axis.ticks.length = unit(7, "pt"),

axis.line = element_line(color = "black"),

axis.text = element_text(color= "black", size = 20),

axis.title = element_blank() )

pdf(paste0("corr_analysis_ApHa/",species[i], "_Eigengenes_v_Titer.pdf"))

plot(line_plot)

dev.off()

# plot legend alone

# use a dataframe like dat but with NAs for x and y values

emptydat <- data.frame(sampleTiter = rep(NA, nrow(dat)),

Module = dat$Module,

value = rep(NA, nrow(dat)) )

legend_plot0 <- ggplot(emptydat, aes(x = sampleTiter, y = value, color = Module)) +

geom_line(na.rm = T, size = 0.5) +

scale_color_manual(values = MEcol)

legend_plot <- legend_plot0 + theme(plot.title = element_blank(),

panel.background = element_blank(),

panel.grid.major = element_blank(),

panel.grid.minor = element_blank(),

axis.line = element_blank(),

axis.text = element_blank(),

axis.title = element_blank(),

axis.ticks = element_blank(),

legend.title = element_blank(),

legend.key = element_rect(fill = NA),

legend.text = element_text(size = 20))

pdf(paste0("corr_analysis_ApHa/",species[i], "_legend.pdf"))

plot(legend_plot)

dev.off()

}

## correlate individual genes with titer, calculate p-values of correlation

ApTiterCor <- cor(t(rldApFilt), datTraits, use = "p")

ApTiterCorP <- corPvalueStudent(ApTiterCor, nSamples = 15)

ApTiterSignificance <- data.frame(correlation = ApTiterCor[,1],

p.val = ApTiterCorP[,1])

HaTraitsCor <- cor(t(rldHaFilt), datTraits, use = "p")

HaTraitsCorP <- corPvalueStudent(HaTraitsCor, nSamples = 15)

HaTraitsSignificance <- data.frame(titer.correlation = HaTraitsCor[,1],

titer.p.val = HaTraitsCorP[,1])

# load deseq2 results tables and format

resOrderedAp <- read.csv("resOrderedAp.csv",

quote = "\"", header = T, stringsAsFactors = F)

rownames(resOrderedAp) <- resOrderedAp[,1 ]

resOrderedAp <- resOrderedAp[,-1 ]

resOrderedHa <- read.csv("resOrderedHA.csv",

quote = "\"", header = T, stringsAsFactors = F)

rownames(resOrderedHa) <- resOrderedHa[,1 ]

resOrderedHa <- resOrderedHa[,-1]

# remove genes not included in the correlation analysis

resOrderedApFilt <- resOrderedAp[rownames(resOrderedAp) %in% names(rowClustCutCol), ]

resOrderedHaFilt <- resOrderedHa[rownames(resOrderedHa) %in% names(colClustCutCol), ]

# make sure gene order matches between resOrderedFilt, ClustCol, and TraitsCor

# are there any rows that don't match?

any(!(rownames(resOrderedApFilt) == names(rowClustCutCol)))

any(!(rownames(resOrderedApFilt) == row.names(ApTiterCor)))

any(!(rownames(resOrderedHaFilt) == names(colClustCutCol)))

any(!(rownames(resOrderedHaFilt) == row.names(HaTraitsCor)))

any(!(names(colClustCutCol) == row.names(HaTraitsCor)))

# order resOrderedXXFilt to match colClustCol

resOrderedHaFilt <- resOrderedHaFilt[match(names(colClustCutCol), rownames(resOrderedHaFilt)), ]

any(!(rownames(resOrderedHaFilt) == row.names(HaTraitsCor)))

any(!(rownames(resOrderedHaFilt) == row.names(HaTraitsCor)))

resOrderedApFilt <- resOrderedApFilt[match(names(rowClustCutCol), rownames(resOrderedApFilt)), ]

any(!(rownames(resOrderedApFilt) == row.names(ApTiterCor)))

any(!(rownames(resOrderedApFilt) == row.names(ApTiterCor)))

# assign names to modules

modNamesAp <- substring(names(MEsAp), 3)

modNamesHa <- substring(names(MEsHa), 3)

# order modules by their significance for titer

modOrderAp <- order(-abs(cor(MEsAp, sampleTiter, use = "p")))

modOrderHa <- order(-abs(cor(MEsHa, datTraits[,1], use = "p")))

## construct an initial summary table for A. pisum

geneInfoAp0 = data.frame(resOrderedApFilt,

moduleColor = rowClustCutCol,

GS.sampleTiter = ApTiterCor,

p.GS.sampleTiter = ApTiterCorP)

# add module membership information to the table in order of significance for titer

for (i in 1:ncol(MMAp)) {

oldNames = names(geneInfoAp0)

geneInfoAp0 = data.frame(geneInfoAp0,

MMAp[, modOrderAp[i]],

MMPvalueAp[, modOrderAp[i]])

names(geneInfoAp0) = c(oldNames, paste("MM.", modNamesAp[modOrderAp[i]], sep=""),

paste("p.MM.", modNamesAp[modOrderAp[i]], sep=""))

}

# save the results

write.table(geneInfoAp0, file = paste0("CorrelationAnalysis_Ap.txt"), quote=F, sep="\t")

## make a similar table for Hamiltonella

geneInfoHa0 = data.frame(resOrderedHaFilt,

moduleColor = colClustCutCol,

GS.sampleTiter = HaTraitsCor[,1],

p.GS.sampleTiter = HaTraitsCorP[,1])

# add module membership information to the table in order of significance for titer

for (i in 1:ncol(MMHa)) {

oldNames = names(geneInfoHa0)

geneInfoHa0 = data.frame(geneInfoHa0,

MMHa[, modOrderHa[i]],

MMPvalueHa[, modOrderHa[i]])

names(geneInfoHa0) = c(oldNames, paste("MM.", modNamesHa[modOrderHa[i]], sep=""),

paste("p.MM.", modNamesHa[modOrderHa[i]], sep=""))

}

# save the results

write.table(geneInfoHa0, file = paste0("CorrelationAnalysis_Ha.txt"), quote=F, sep="\t")

# rename some things

geneInfoAp <- geneInfoAp0

geneInfoHa <- geneInfoHa0

##Prepare GO-Enrichment analysis

# put aphid correlation module names into a vector

intModules = unique(rowClustCutCol)

# prepare an empty list to place entrez IDs for each module in

intModulesList <- list()

# pick out entrez numbers for every gene in each module of interest

for (module in intModules) {

# select module probes

modGenes = (rowClustCutCol==module)

# get their entrez ID

modGenes = names(rowClustCutCol)[modGenes]

# save objects in a list

intModulesList[[module]] <- modGenes

}

for(item in names(intModulesList)){

table<-as.data.frame(cbind(rownames(resOrderedApFilt),

ifelse(rownames(resOrderedApFilt)%in%intModulesList[[item]], 1, 0)))

write.table(file=paste("corr_module_membership/Corr_Ap_module_",item,".txt", sep=""),

quote=F, row.names = F, col.names=F,sep="\t", table)}

# put Hamiltonella correlation module names into a vector

intModules = unique(colClustCutCol)

# prepare an empty list to place entrez IDs for each module in

intModulesList <- list()

# pick out entrez numbers for every gene in each module of interest

for (module in intModules) {

# select module probes

modGenes = (colClustCutCol==module)

# get their entrez ID

modGenes = names(colClustCutCol)[modGenes]

# save objects in a list

intModulesList[[module]] <- modGenes

}

for(item in names(intModulesList)){

table<-as.data.frame(cbind(rownames(resOrderedHaFilt),

ifelse(rownames(resOrderedHaFilt)%in%intModulesList[[item]], 1, 0)))

write.table(file=paste("corr_module_membership/Corr_Ha_module_",item,".txt", sep=""),

quote=F, row.names = F, col.names=F,sep="\t", table)}
